# Inhibiting the anaphase promoting complex/cyclosome induces a metaphase arrest and cell death in multiple myeloma cells

**DOI:** 10.18632/oncotarget.6768

**Published:** 2015-12-26

**Authors:** Susanne Lub, Anke Maes, Ken Maes, Kim De Veirman, Elke De Bruyne, Eline Menu, Karel Fostier, Alboukadel Kassambara, Jérôme Moreaux, Dirk Hose, Xavier Leleu, Randall W. King, Karin Vanderkerken, Els Van Valckenborgh

**Affiliations:** ^1^ Laboratory of Hematology and Immunology, Myeloma Center Brussels, Vrije Universiteit Brussel, Brussels, Belgium; ^2^ Department of Biological Hematology, CHU Montpellier, Montpellier, France; ^3^ Institute of Human Genetics, CNRS-UPR1142, Montpellier, France; ^4^ University of Montpellier 1, UFR de Médecine, Montpellier, France; ^5^ Medizinische Klinik, Universitätsklinikum Heidelberg, Heidelberg, Germany; ^6^ Service des maladies du sang, Hôpital Huriez, CHRU, Lille, France; ^7^ Department of Cell Biology, Harvard Medical School, Boston, Massachusetts, USA

**Keywords:** multiple myeloma, high-risk, anaphase promoting complex/cyclosome, Cdc20, proTAME

## Abstract

The anaphase promoting complex/cyclosome (APC/C) is an ubiquitin ligase involved in cell cycle. During the metaphase-anaphase transition the APC/C is activated by Cdc20. The aim of this study is to elucidate the importance and therapeutic potential of APC/C and its co-activator Cdc20 in multiple myeloma (MM). Gene expression analysis revealed that Cdc20 was expressed at higher levels in gene expression-based high-risk MM patients. Moreover, high Cdc20 expression correlated with poor prognosis. Treatment of human myeloma cell lines with proTAME, an APC/C inhibitor, resulted in an accumulation of APC/C^Cdc20^ substrate cyclin B1 and an accumulation of cells in metaphase. Moreover we observed a significant dose-dependent decrease in viability and increase in apoptosis in MM cells upon proTAME treatment. The induction of apoptosis was accompanied with caspase 3, 8, 9 and PARP cleavage. A similar metaphase arrest and induction of apoptosis were obtained with specific knockdown of Cdc20. In addition, we demonstrated the accumulation of Bim was partially responsible for the observed cell death. Combining proTAME with another APC/C inhibitor apcin or the alkylating agent melphalan resulted in enhanced anti-MM activity. This study suggests that the APC/C and its co-activator Cdc20 could be a new and promising target especially in high-risk MM patients.

## INTRODUCTION

Multiple myeloma (MM) is a plasma cell cancer characterized by clonal proliferation of plasma cells in the bone marrow (BM). A great improvement in the survival of MM patients was achieved the past decade due to the development of novel treatment options including novel agents and autologous stem cell transplantation (ASCT) for younger patients. These novel agents include the proteasome inhibitor bortezomib and the immunomodulatory drug lenalidomide. Nevertheless, these agents are not curative for the majority of patients and therefore investigation of novel targets to enhance therapy is necessary [1, 2].

The ubiquitin-proteasome system (UPS) is highly regulated and controls different protein functions and processes such as proliferation, metabolism and apoptosis by targeting cellular proteins for degradation. The ubiquitination process is dependent on ATP and requires the E1, E2 and E3 enzymes. First theubiquitin-activating enzyme E1 activates ubiquitin which is then delivered to the E2 ubiquitin-conjugating enzyme. The E2 subsequently forms a complex with the ubiquitin-ligase E3 and the target protein. The E3 transfers ubiquitin to the substrate to form a polyubiquitin chain, targeting the protein for degradation by the proteasome [3]. The success of the proteasome inhibitor bortezomib in the treatment of MM highlights the importance of the UPS in this disease. Selective targeting of more disease-specific components of the UPS might result in more effective treatment [4, 5]. There is an increasing interest in ubiquitin ligases as targets since they play a key role in the UPS by determining the protein to be ubiquitylated and thereby controlling cell function in a very specific way.

The anaphase promoting complex/cyclosome (APC/C) is a 13-subunit ubiquitin ligase protein complex that controls the cell cycle. The regulation of the APC/C is dependent on 2 co-activators, Cdc20 during the metaphase-anaphase transition and Cdh1 during mitotic exit and early G1-phase. Binding of the co-activator to the complex leads to the recruitment of substrates which will be targeted for proteasomal degradation. Key targets of APC/C^Cdh1^ during mitotic exit and G1 maintenance are Skp2 and Ets2 leading to an increase in the levels of p27^Kip1^, p21^Cip1^ and cyclin D1 that are necessary to maintain the G1 phase [6]. Targets of the APC/C^Cdc20^ complex are the cell cycle proteins cyclin B1 and securin. Their proteasomal degradation leads to the onset of the anaphase and mitotic exit. A major regulator of the APC/C^Cdc20^ complex is the spindle assembly checkpoint (SAC). The SAC delays chromosome segregation until all kinetochores become properly attached to the mitotic spindle, then the APC/C^Cdc20^ is activated [7].

Several studies suggest that the APC/C and its co-activator Cdc20 could be potential new therapeutic targets in cancer. Cdc20 overexpression has been correlated with poor prognosis in several solid cancers [8–14]. Knockdown of Cdc20 in various cancer cell lines caused mitotic arrest, cell death and increased sensitivity to chemotherapeutics and radiation [15–17]. Small molecule inhibitors of the APC/C called proTAME and apcin, have recently been discovered. ProTAME is a cell permeable prodrug that is converted to TAME (Tosyl-L-Arginine Methyl Ester) by intracellular esterases. TAME structurally mimics the IR-tail of the co-activators and therefore binds to APC/C, blocking the interaction of Cdc20 or Cdh1 with the APC/C [18]. Apcin is a small molecule that prevents substrate recognition by binding to Cdc20 [19]. In MM little is known about the APC/C and its co-activators. So far it has been shown that Cdc20 and the different components of the SAC are expressed in human MM cell lines (HMCLs) [20]. Our aim is to elucidate the importance and therapeutic potential of APC/C and its co-activator Cdc20 in MM.

## RESULTS

### Gene expression and prognostic value of Cdc20 and Cdh1 in MM patients

**Figure 1:**
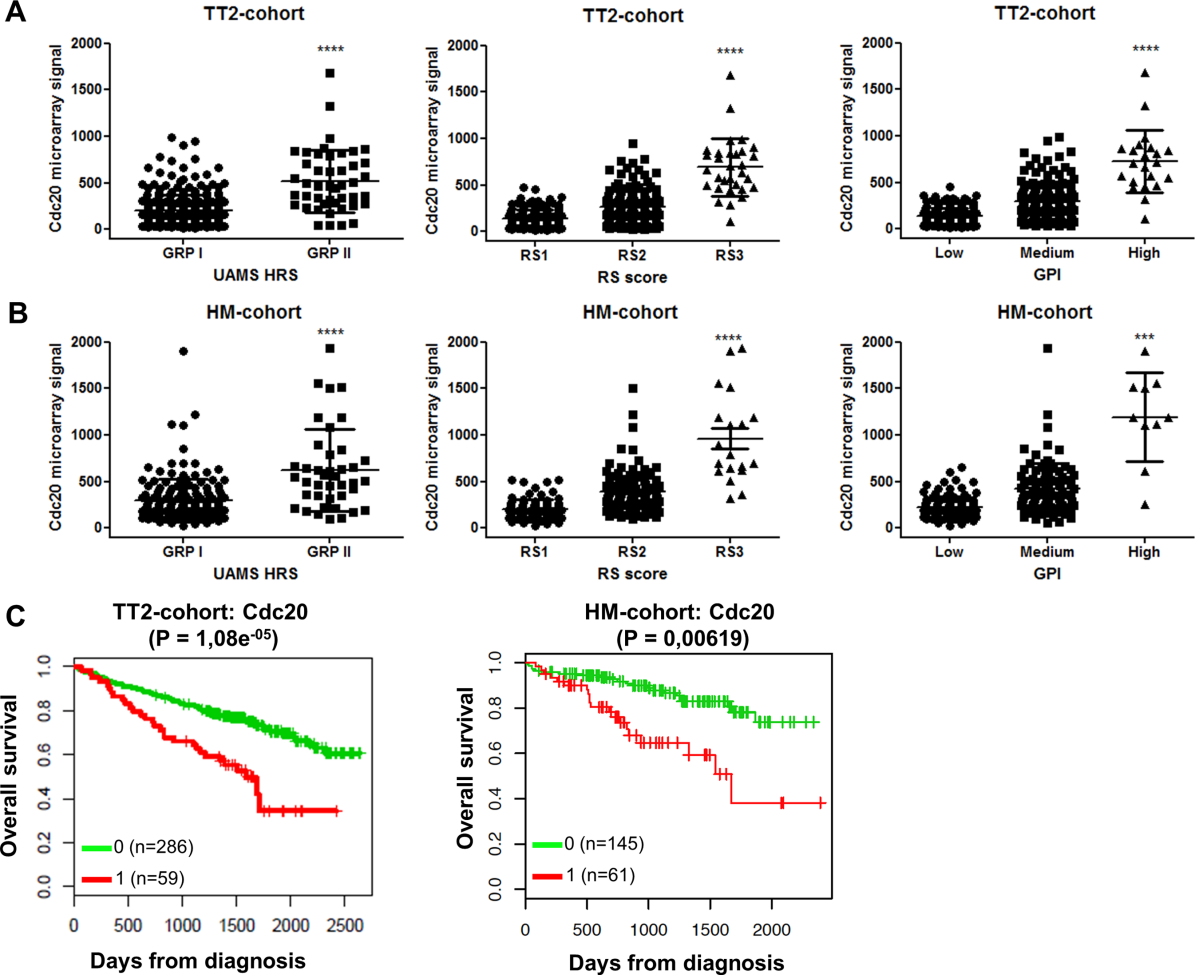
Cdc20 expression levels and prognostic value in MM patients (**A**–**B**) Association of Cdc20 expression levels in MM patients with gene expression-based high-risk scores in TT2-cohort and HM-cohort. The high-risk groups are compared to the overall mean expression in all groups. ***indicates *p*-value is < 0.001 and ****p-value is < 0.0001 (student *t*-test). (**C**) The overall survival in the TT2-cohort and HM-cohort for low (green) or high (red) Cdc20 expression in MM patients.

We assessed the distribution of Cdc20 expression in primary MM cells using three gene expression-based high-risk scores namely the RS, GPI and UAMS HRS scores [21–23]. In two independent MM patient cohorts we observed a significant higher Cdc20 expression in all high-risk groups (Figure 1A–1B). In addition, high Cdc20 expression correlated with a significant inferior overall survival in MM patients in both cohorts (Figure 1C). However there was no association between Cdh1 expression and gene expression-based risk scores in MM patients (Supplementary Figure 1A–1B). Low Cdh1 expression correlated with poor prognosis in MM in the TT-2 cohort but had no prognostic value in the HM-cohort (Supplementary Figure 1C). Next we evaluated the microarray expression level of Cdc20 and Cdh1 in HMCLs and observed a rather constant and low expression of Cdh1 while Cdc20 expression was higher and more variable (Supplementary Figure 1D). Moreover the expression of Cdc20 in primary MM cells correlated with proliferation measured as the plasma cell proliferation index with PI staining (*n =* 101, *r*_s_ = 0,416, *P* < 0.001) (Supplementary Figure 2).

Gene set enrichment analysis was performed comparing gene expression profiles of MM cells of patients with high or low Cdc20 or Cdh1 expression. A list of the top 10 GSEA pathways enriched in Cdc20 high, Cdc20 low, Cdh1 high and Cdh1 low MM patients can be found in Tables 1 and 2. MM cells of patients with a high Cdc20 expression showed a significant enrichment in genes associated with proliferation, while MM cells of patients with a low Cdc20 expression had a significant enrichment in genes under-expressed in the proliferation subgroup of the MM molecular classification (Supplementary Figure 3 and 4 and Supplementary Tables 1–4). Patients with high Cdh1 expression were characterized by a significant enrichment of genes related to mature bone marrow plasma cells and JAK/STAT signaling. Interestingly, patients with low Cdh1 expression showed a significant enrichment of MYC target genes (Supplementary Figure 5 and 6 and Supplementary Tables 5–7).

**Table 1:** GSEA pathways significantly enriched in MM patients with high or low Cdc20 expression

	Size	Es	Nes	*p*-value
**GSEA pathways significantly enriched in MM patients with high Cdc20 expression**				
pujana_chek2_pcc_network	414	−0.68	−2.66	< 0.0001
zheng_glioblastoma_plasticity_up	207	−0.69	−2.65	< 0.0001
shedden_lung_cancer_poor_survival_a6	319	−0.78	−2.62	< 0.0001
pujana_brca2_pcc_network	280	−0.75	−2.62	< 0.0001
tarte_plasma_cell_vs_plasmablast_dn	194	−0.77	−2.61	< 0.0001
wong_embryonic_stem_cell_core	190	−0.77	−2.60	< 0.0001
reactome_cell_cycle	251	−0.72	−2.58	< 0.0001
vecchi_gastric_cancer_early_up	313	−0.73	−2.58	< 0.0001
patil_liver_cancer	473	−0.61	−2.58	< 0.0001
lindgren_bladder_cancer_cluster_3_up	218	−0.75	−2.58	< 0.0001
**GSEA pathways significantly enriched in MM patients with low Cdc20 expression**				
reactome_inhibition_of_voltage_gated_ca2_channels_via_gbeta_gamma_subunits	21	0.63	1.85	< 0.0001
zhan_multiple_myeloma_pr_dn	32	0.62	1.81	0.023
vilimas_notch1_targets_up	47	0.49	1.75	0.004
reactome_inwardly_rectifying_k_channels	26	0.58	1.73	0.004
biocarta_cardiacegf_pathway	15	0.62	1.73	0.008
sumi_hnf4a_targets	27	0.54	1.72	0.013
kondo_prostate_cancer_with_h3k27me3	129	0.45	1.70	0.002
meissner_brain_hcp_with_h3_unmethylated	29	0.56	1.69	0.008
reactome_nuclear_receptor_transcription_pathway	37	0.50	1.69	0.012

**Table 2:** GSEA pathways significantly enriched in MM patients with high or low Cdh1 expression

	Size	Es	Nes	*p*-value
**GSEA pathways significantly enriched in MM patients with high Cdh1 expression**				
reactome_growth_hormone_receptor_signaling	22	−0.58	−1.88	0.004
mueller_common_targets_of_aml_fusions_dn	23	−0.60	−1.80	0.004
kegg_jak_stat_signaling_pathway	132	−0.41	−1.80	< 0.0001
mccabe_hoxc6_targets_cancer_up	22	−0.58	−1.75	0.006
kuuselo_pancreatic_cancer_19q13_amplification	15	−0.62	−1.74	0.012
moreaux_multiple_myeloma_by_taci_up	288	−0.42	−1.72	< 0.0001
mueller_methylated_in_glioblastoma	39	−0.52	−1.73	< 0.0001
reactome_effects_of_pip2_hydrolysis	19	−0.58	−1.73	0.011
mcmurray_tp53_hras_cooperation_response_up	21	−0.57	−1.72	0.010
pomeroy_medulloblastoma_desmoplasic_vs_classic_dn	42	−0.48	−1.72	0.002
**GSEA pathways significantly enriched in MM patients with low Cdh1 expression**				
reactome_pyruvate_metabolism_and_citric_acid_tca_cycle	18	0.73	2.07	0.006
schuhmacher_myc_targets_up	42	0.64	2.00	0.002
gazda_diamond_blackfan_anemia_progenitor_dn	34	0.57	1.97	< 0.0001
chow_rassf1_targets_dn	15	0.74	1.95	< 0.0001
zhan_multiple_myeloma_cd1_up	39	0.81	1.79	0.020
krige_amino_acid_deprivation	28	0.72	1.79	0.026
chiang_liver_cancer_subclass_unannotated_dn	93	0.50	1.78	0.022
zhan_multiple_myeloma_cd1_vs_cd2_up	57	0.73	1.77	0.026
schlosser_myc_targets_and_serum_response_up	24	0.65	1.75	0.018
shaffer_irf4_targets_in_myeloma_vs_mature_b_lymphocyte	70	0.50	1.73	0.013

### Pharmacological inhibition of the APC/C with proTAME results in a metaphase arrest and reduced viability of MM cells

**Figure 2:**
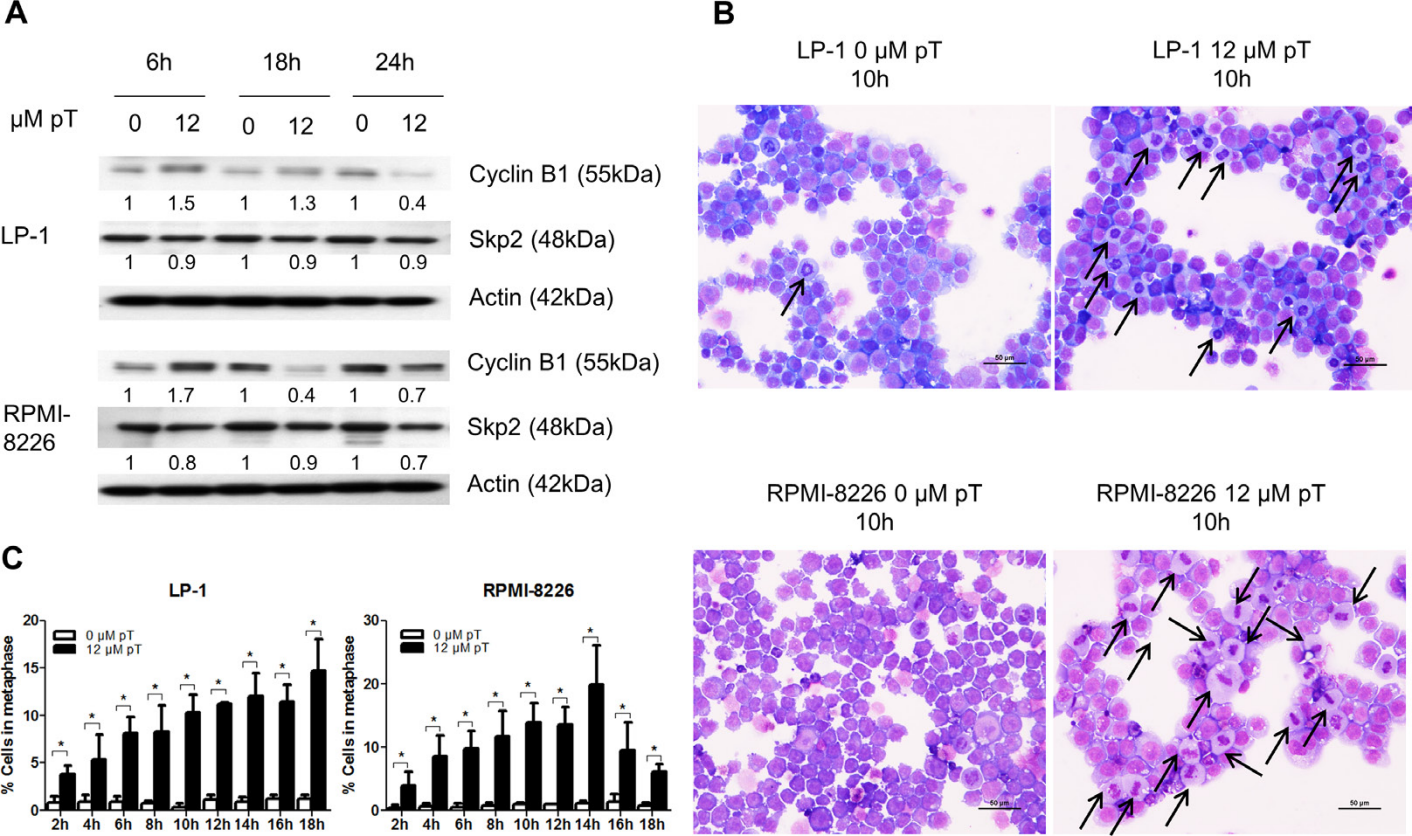
Pharmacological inhibition of the APC/C with proTAME results in a metaphase arrest (**A**) LP-1 and RPMI-8226 cells were treated with 12 μM proTAME (pT) for 6, 18 and 24 hours. Western blot analysis was performed using cyclin B1, Skp2 and β-actin antibodies. This result is representative for 3 independent experiments. The pixel densities of proteins are normalized to β-actin and relative to control. (**B**) LP-1 and RPMI-8226 cells were treated with 12 μM proTAME and every 2 hours until 18 hours May-Grünwald Giemsa stained cytospins were made. Arrows point out the MM cells in metaphase. Scale bar measures 50 μm. (**C**) Quantification of the amount of cells in metaphase. Results shown in each graph are the mean of 3 independent experiments ± SD. *means the *p*-value is < 0.05 (Mann-Whitney *U*-test).

To assess if the APC/C could be a potential target in MM we used the APC/C inhibitor proTAME. We first examined protein levels of substrates APC/C^Cdc20^ (cyclin B1) and APC/C^Cdh1^ (Skp2) after 6, 18 and 24 hours proTAME treatment (Figure 2A). We observed an increase in cyclin B1 at early time points. Skp2 levels however were not affected by proTAME treatment. This suggests that proTAME may preferentially inhibit APC/C^Cdc20^ activity in MM cells.

Since the APC/C^Cdc20^ is involved in the metaphase-anaphase transition we investigated if inhibition of the APC/C could lead to an arrest of cells in the metaphase. May-Grünwald Giemsa stained cytospins of proTAME treated LP-1 and RPMI-8226 cells were analyzed using light microscopy (Figure 2B). Quantification of the percentage of cells in metaphase indicated a significant increase when the cell lines were treated with proTAME at each time point (Figure 2C).

**Figure 3:**
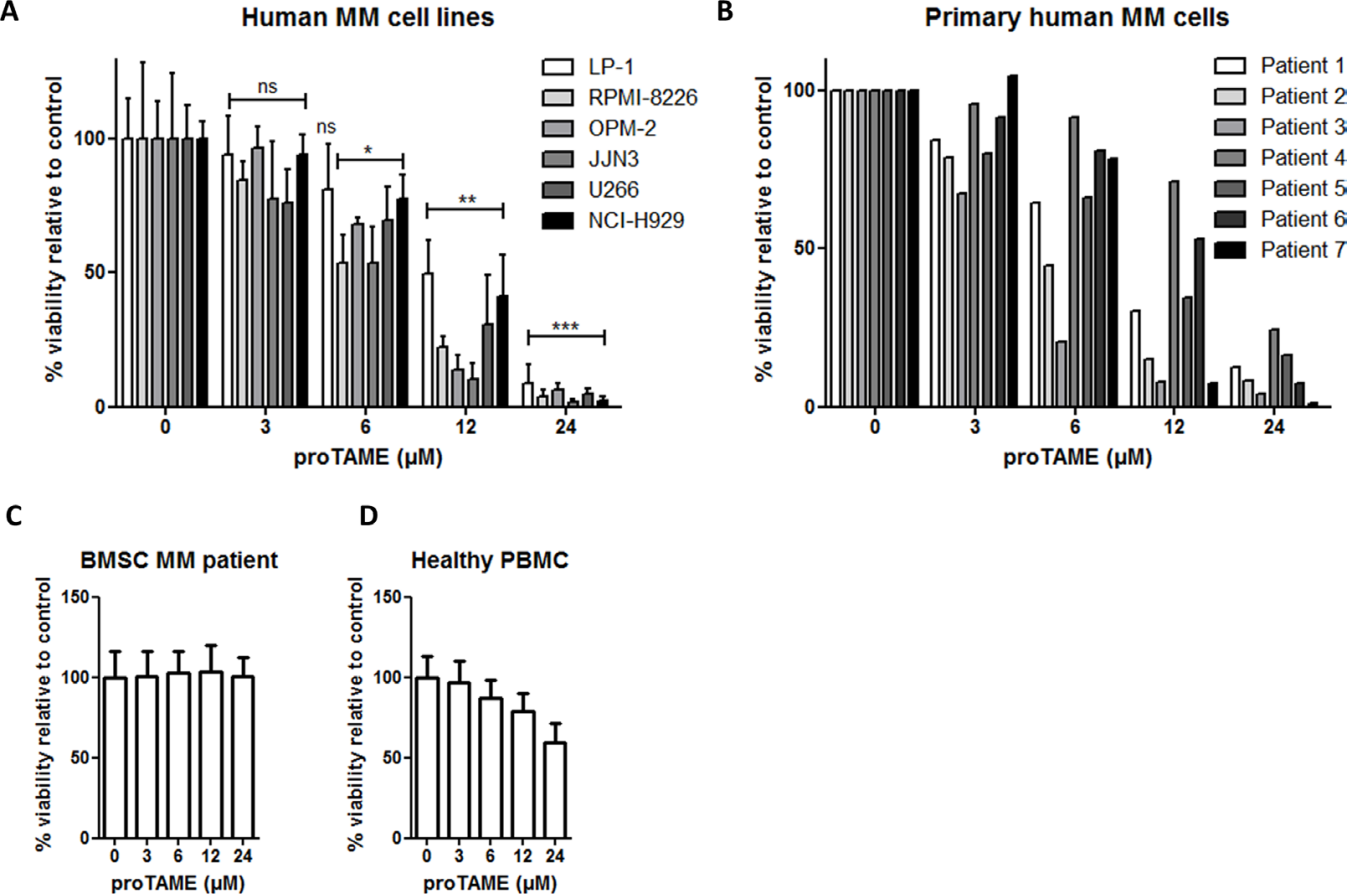
Pharmacological inhibition of the APC/C with proTAME results in a decreased viability of MM cells (**A**) LP-1, RPMI-8226, JJN3, OPM-2, U266, NCI-H929, (**B**) primary human MM cells, (**C**) BMSC of MM patients and (**D**) healthy peripheral blood mononuclear cells were treated with 3, 6, 12 and 24 μM proTAME and after 24 hours viability was measured with CellTiter-Glo assay. Results shown are the mean of 4 independent experiments for HMCL, 2 for BMSC of MM patients and 3 for PBMC ± SD. *means the *p*-value is < 0.05, **means the *p*-value is < 0.001, ***means the *p*-value is < 0.0001 and represents a significant decrease compared to control (One-way ANOVA). For the primary human MM cells the result of each patient is shown individually.

As a mitotic arrest can lead to cell death we further investigated the effect of proTAME on the viability of MM cells. The HMCLs LP-1, RPMI-8226, JJN3, OPM-2, U266 and NCI-H929 were treated with proTAME and viability was measured 24 hours later (Figure 3A). We observed a dose-dependent decrease in the viability in all HMCLs. LP-1 was the least sensitive cell line with an IC50 of 12.1 μM and JJN3 was the most sensitive with an IC50 of 4.8 μM (Supplementary Table 8). In addition we tested proTAME on primary samples from 7 myeloma patients and observed again a dose-dependent reduction of the viability (Figure 3B). The IC50 varied among the different patients, ranging from 2.8 to 20.3 μM (Supplementary Table 8). Patient characteristics can be found in Supplementary Table 9. To investigate if proTAME influences the viability of cells from the BM microenvironment, we treated bone marrow stromal cells (BMSC) from different MM patients (Figure 3C) and observed that proTAME did not affect their viability. Moreover we treated peripheral blood mononuclear cells (PBMCs) of 3 healthy persons with proTAME (Figure 3D). ProTAME decreased the viability of PBMCs in a dose dependent way with an IC50 of 73,6 μM which is higher compared to the IC50 of MM cells (3,7–26,3x higher than MM patient cells and 6,1–15,3x higher as HMCLs).

### proTAME is still active in the presence of the BM microenvironment

**Figure 4:**
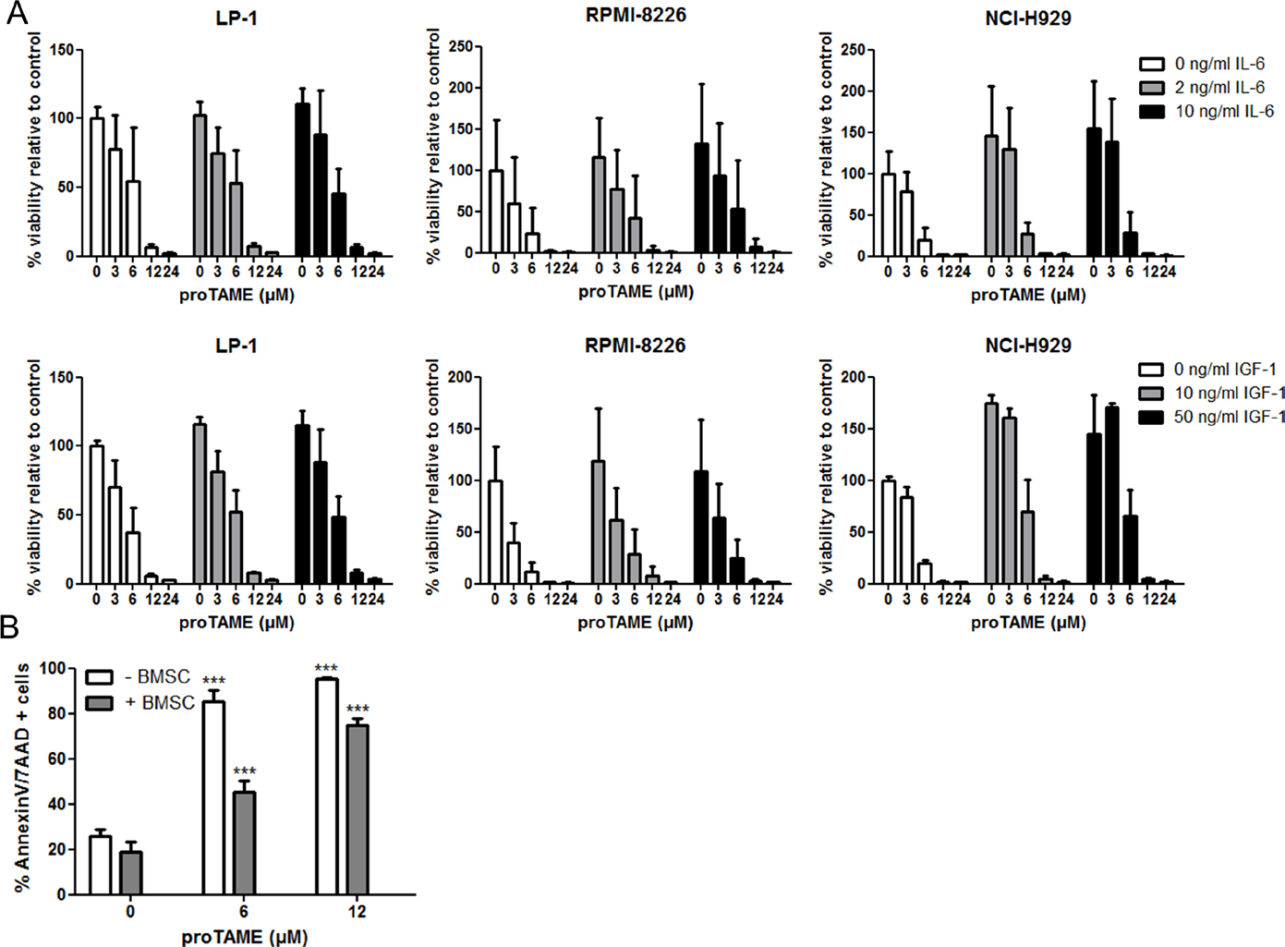
The effect of proTAME on MM cells in the presence of growth factors or BMSCs (**A**) HMCL LP-1, RPMI-8226 and NCI-H929 were cultured in serum free conditions and treated with 0, 3, 6, 12 and 24 μM proTAME in combination with IL-6 or IGF-1. After 24 hours the viability was determined with the CellTiter-Glo assay. (**B**) RPMI-8226 cells were cultured in serum free conditions and treated with 0, 6, 12 μM proTAME in the presence or absence of human healthy BMSCs. After 24 hours apoptosis was examined with Annexin V/7AAD flow cytometry staining. Results shown in the graphs are the mean of 3 independent experiments ± SD. ***means the *p*-value is < 0.0001 (One-way ANOVA) and indicates a significant increase compared to control (0 μM proTAME).

The BM microenvironment is an important source of MM survival and growth factors such as IL-6 and IGF-1. Here we tested if IL-6 and IGF-1 could abrogate the effect of proTAME on HMCLs by investigating the viability of LP-1, RPMI-8226 and NCI-H929 cells treated with proTAME with or without IL-6 or IGF-1. In all these MM cell lines IL-6 and IGF-1 induced an increased survival at both concentrations tested. Although in some conditions IL-6 and IGF-1 slightly diminished the effect of proTAME, at higher concentrations the effect of proTAME was not abrogated by these cytokines (Figure 4A). We also co-cultured the RPMI-8226 cells with healthy human bone marrow stromal cells (BMSCs) and treated them with proTAME. Although BMSCs reduced the percentage of apoptotic cells, proTAME was still able to significantly induce apoptosis in MM cells in the presence of BMSCs (Figure 4B).

### Pharmacological inhibition of the APC/C with proTAME induces apoptosis in MM cells and is partially mediated by Bim and phosphorylation of Bcl-2 and Bcl-xL

**Figure 5:**
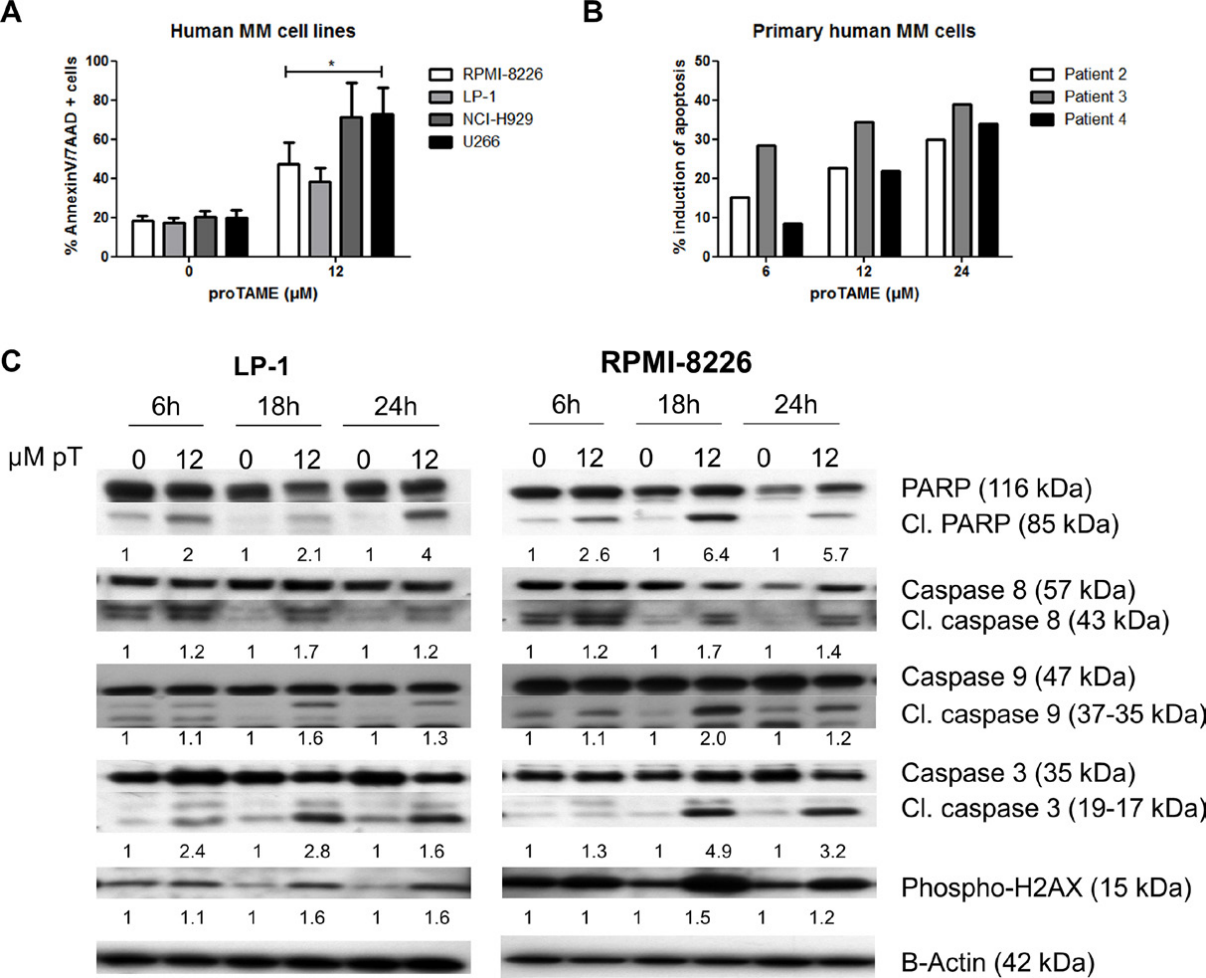
Pharmacological inhibition of the APC/C with proTAME induces apoptosis in MM cells (**A**) LP-1, RPMI-8226, NCI-H929 and U266 cells weretreated with 12 μM proTAME and after 48 hours apoptosis was examined with Annexin V/7AAD flow cytometry staining. Results shown in the graph are the mean of 4 independent experiments ± SD,*means the *p*-value is < 0.05 and represents a significant increase compared to control (Mann-Whitney *U*-test). (**B**) Primary human MM cells were treated with indicated concentrations of proTAME and after 24 hours the induction of apoptosis was calculated with Annexin V/7AAD flow cytometry staining. The result of each patient is shown individually. (**C**) LP-1 and RPMI-8226 cells were treated with 12 μM proTAME for 6, 18 and 24 hours. Western blot was performed using caspase 3, 8, 9, PARP, phospho-H2AX and β-actin antibodies. The pixel densities of proteins were normalized to β-actin and relative to control. This result is representative for 3 independent experiments.

Next we explored if APC/C inhibition could induce apoptosis in 4 cell lines. A significant increase in the number of apoptotic cells was found in each cell line after treatment with proTAME (Figure 5A). This was confirmed by an induction of apoptosis in primary samples from 3 myeloma patients (Figure 5B).

**Figure 6:**
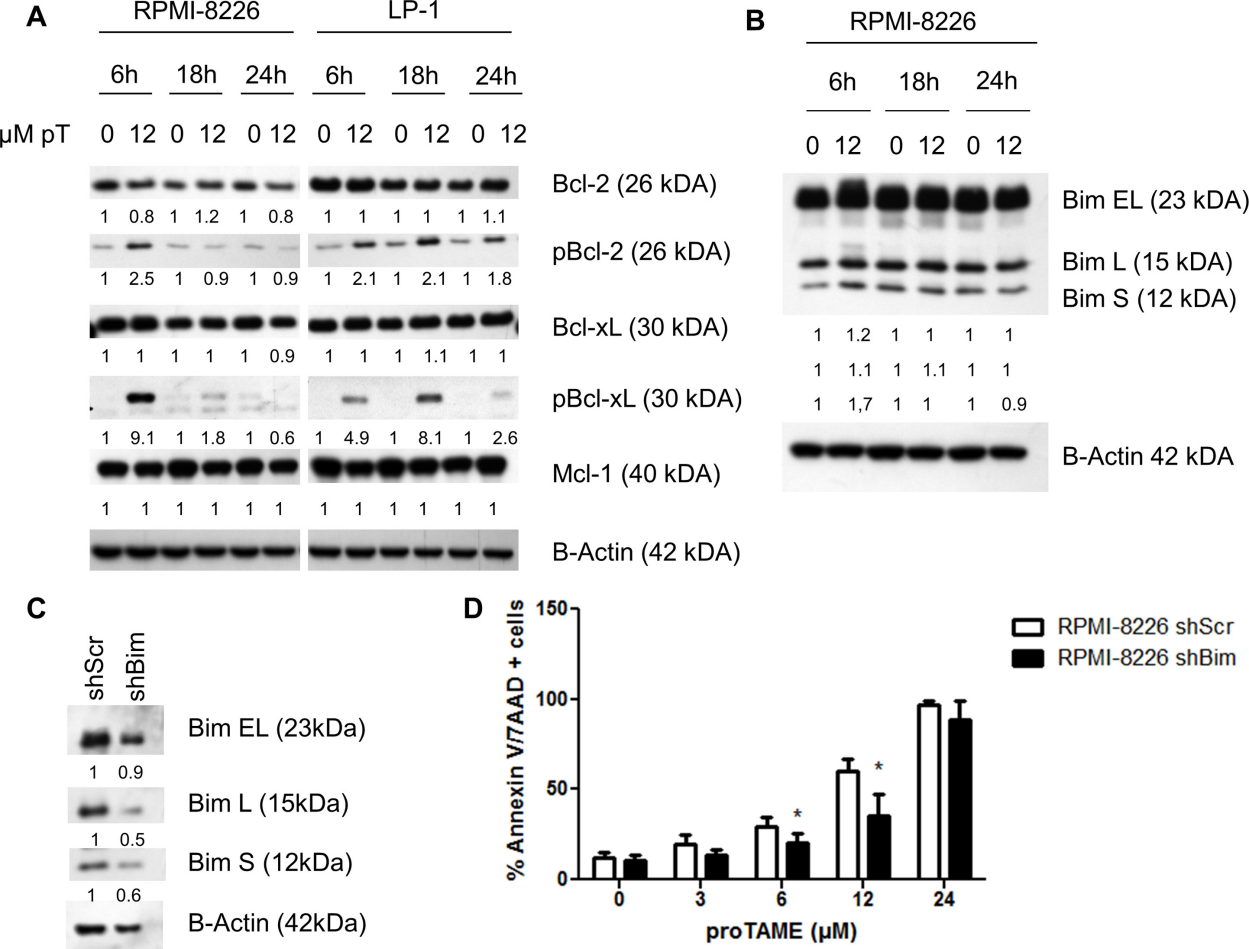
proTAME treatment induces Bim and phosphorylation of Bcl-2 and Bcl-xL (**A** and **B**) LP-1 and RPMI-8226 cells were treated with 12 μM proTAME for 6, 18 and 24 hours. Western blot was performed using Bcl-2, pBcl-2, Bcl-xL, pBcl-xL, Mcl-1, Bim and β-actin antibodies. The pixel densities of proteins were normalized to β-actin and relative to control. This result is representative for 3 independent experiments. (**C**) Bim was knocked down in the RPMI-8226 using shRNA. These cells were treated with 0, 3, 6, 12 and 24 μM proTAME and after 48 hours apoptosis was measured with Annexin V/7AAD flow cytometry staining. Results shown in each graph are the mean of 4 independent experiments ± SD,*means the *p*-value is < 0.05 and indicates a significant difference compared to RPMI-8226shScr (Mann-Whitney *U*-test) (**D**).

The apoptotic pathway was further investigated in LP-1 and RPMI-8226 cells by western blot. We observed cleavage of PARP, caspase 3, 8 and 9 beginning after 6 hours of proTAME treatment in both cell lines. Moreover, we observed a phosphorylation of H2AX after proTAME treatment (Figure 5C). Looking at Bcl-2 family proteins, no clear difference was observed in total Bcl-2, Bcl-xL and Mcl-1 however phosphorylation of Bcl-2 and Bcl-xL was clearly induced at earlier time points in HMCLs treated with proTAME (Figure 6A). In addition, Bim was increased when RPMI-8226 cells were incubated for 6 hours with proTAME (Figure 6B). To investigate the involvement of Bim in the induced apoptosis, Bim was silenced in RPMI-8226 cells with shRNA. Bim silencing was confirmed by western blot analysis of cells transduced with a shBim lentiviral vector compared to shScrambled transduced cells (Figure 6C). Reduction of Bim expression in RPMI-8226 cells significantly reduced the effect of proTAME, implying that Bim is at least partially responsible for the observed induction of apoptosis by proTAME (Figure 6D). LP-1 cells were not investigated for the involvement of Bim as Bim is not expressed in this cell line [24].

### Knockdown of Cdc20 results in a metaphase arrest and apoptosis in MM cells

**Figure 7:**
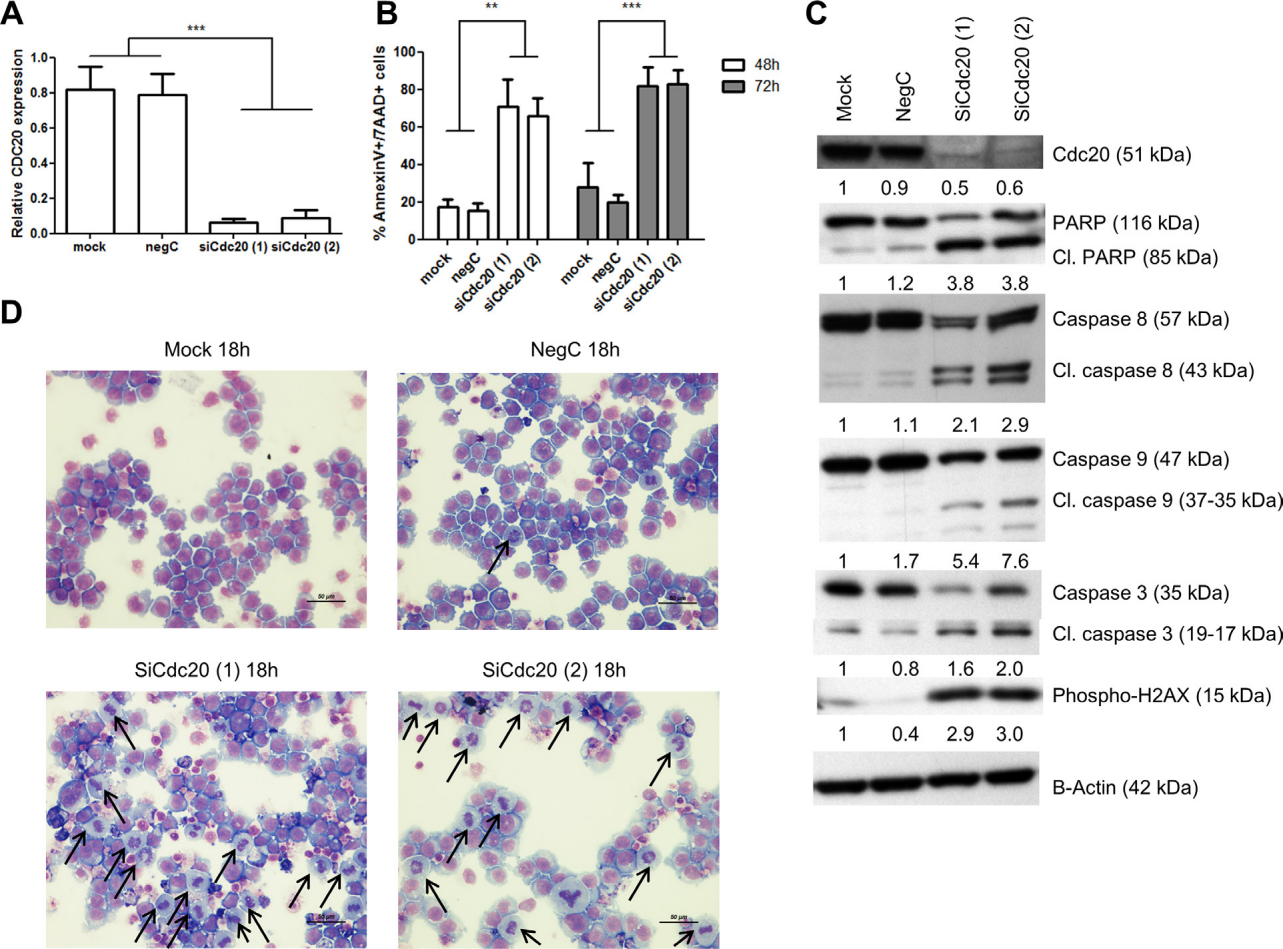
Knockdown of Cdc20 results in a metaphase arrest and cell death RPMI-8226 cells were transfected using lipofectamine RNAiMAX reagent and two different siRNA duplexes against Cdc20. 48 hours after transfection (**A**) Q-PCR was performed. Results shown are the mean of 3 independent experiments ± SD. ***means the *p*-value is < 0.0001 (One-way ANOVA). (**B**) Apoptosis was examined with Annexin V/7AAD flow cytometry staining. Results shown in the graph are the mean of 3 independent experiments ± SD, **and ***mean the *p*-value is respectively < 0.001 and < 0.0001 (One-way ANOVA). (**C**) Western blot was performed using Cdc20, caspase 3, 8, 9, PARP, phospho-H2AX and β-actin antibodies. The pixel densities of proteins were normalized to β-actin and relative to mock. This result is representative for 3 independent experiments. (**D**) 18 hours after transfection May-Grünwald Giemsa stained cytospins were made. Arrows point out the MM cells in metaphase.

To exclude that proTAME has non-specific effects we knocked down Cdc20 with siRNA in the HMCL RPMI-8226. Q-PCR and western blot analysis demonstrated a significant reduction of Cdc20 on respectively mRNA and protein levels. Knockdown of Cdc20 resulted in a significant induction of apoptosis after 48 and 72 h which was accompanied with cleavage of caspase 3, 8, 9 and PARP. Moreover, silencing Cdc20 resulted in phosphorylation of H2AX and accumulation of cells in metaphase (Figure 7A–7D).

### Inhibition of the APC/C with two chemical inhibitors significantly increases MM cell death

**Figure 8:**
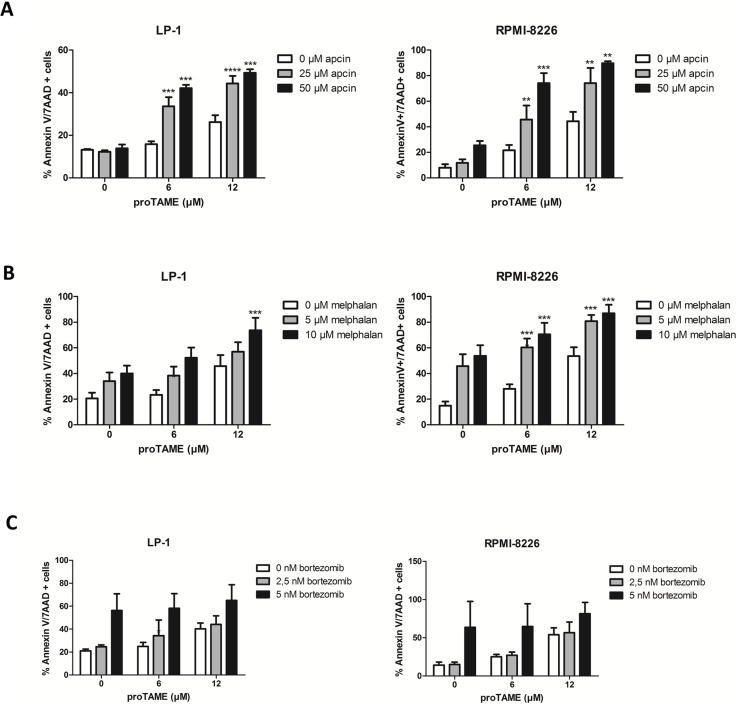
MM cell death is significantly increased when the APC/C is inhibited with two chemical inhibitors or in combination with melphalan LP-1 and RPMI-8226 were treated with proTAME in combination with another APC/C inhibitor apcin (**A**), melphalan (**B**) or bortezomib (**C**). After 48 hours apoptosis was measured with Annexin V/7AAD flow cytometry staining. Results shown in each graph are the mean of at least 3 independent experiments ± SD. **means the *p*-value is < 0.001, ***means the *p*-value is < 0.0001 and represents a significant increase compared to single treatments (One-way ANOVA).

Recently it was described that combining proTAME with apcin leads to a synergistic inhibition of the APC/C [19]. We therefore incubated the HMCLs LP-1 and RPMI-8226 with a combination of proTAME and apcin. Although apcin alone had minor effects on MM cells, the combination of both inhibitors increased apoptosis significantly compared to single treatments (Figure 8A).

We also investigated the combination of proTAME with 2 important drugs used for the treatment of MM patients, namely melphalan and bortezomib. The combination of proTAME with melphalan increased apoptosis significantly compared to single treatments (Figure 8B). However, combining proTAME with bortezomib did not result in an increased apoptosis (Figure 8C) compared to single treatments.

## DISCUSSION

Since the outcome of particularly high-risk patients has not yet improved, investigation of alternative treatment options of those patients is greatly needed [25]. High-risk MM patients are characterized by an overexpression of genes associated with cell cycle and a high labeling index [21, 26–28]. This gives a clear rationale to treat high-risk patients with (targeted) anti-mitotics. In this study we investigated if the APC/C, and its co-activator Cdc20 could be a potential target in MM. Based on three existing gene expression-based high-risk scores [21–23] we observed a higher Cdc20 expression in high-risk MM patient groups. Moreover we observed a correlation between Cdc20 expression in primary MM cells and proliferation. High Cdc20 was associated with poor overall survival in both MM patient cohorts. This is in agreement with other cancer types where an upregulated Cdc20 expression correlated with a poor prognosis [8–14]. Moreover a recent study shows that MM patients with a high expression of BUB1b, Cdc20 and CCNB1/2 had a poor survival outcome in the TT-2 cohort [29]. In addition we showed that MM cells of patients with a high Cdc20 expression showed a significant enrichment in genes associated with proliferation whereas MM cells of patients with low Cdc20 expression have a significant enrichment in genes underexpressed in the proliferation subgroup of the MM molecular classification. Cdh1 expression on the other hand was not associated with gene expression-based high-risk MM. Low Cdh1 expression was associated with poor prognosis in the TT-2 cohort but not in the HM-cohort. Cdh1 has been described as a potential tumor suppressor, since elevated Cdh1 levels inhibited breast tumor growth and depletion of Cdh1 induced proliferation of breast cancer cells [30–32]. We observed that patients with high Cdh1 expression had a significant enrichment of genes related to mature bone marrow plasma cells and JAK/STAT signaling whereas patients with low Cdh1 expression showed a significant enrichment in MYC target genes. From this we concluded that it would be interesting to target the APC/C^Cdc20^ in MM.

In this study we used the APC/C inhibitor proTAME. Previous research demonstrated that proTAME is able to inhibit the interaction of both co-activators with the APC/C in HeLa cells [18]. Inhibition by proTAME efficiently induced a metaphase arrest, which is in agreement with our study. Moreover we observed at early time points a clear increase in cyclin B1, a substrate of the APC/C^Cdc20^, while the APC/C^Cdh1^ substrate Skp2 was not affected. This could be due to the fact that cells arrest in metaphase first by the effect on APC/C^Cdc20^ upon proTAME treatment making it difficult to see effects of proTAME on APC/C^Cdh1^. The lack of cyclin B1 accumulation at later time points could be because of cells dying faster than they can accumulate cyclin B1. By western blot analysis we could indeed observe signs of cell death beginning as early as 6 hours following proTAME treatment. The observed metaphase arrest was followed by a reduction in the viability. The sensitivity of the different HMCLs is variable with an IC50 ranging from 4.8 to 12.1 μM. The variability in sensitivity can be attributed to the difference in proliferation rate between the different cell lines. Primary MM samples were sensitive to proTAME treatment with an IC50 ranging from 2.8 to 20.3 μM, comparable to the IC50 described in HeLA cells [18]. However non-malignant cells of MM patients and healthy PBMCs were not or only slightly (at the highest concentration) affected by proTAME. We furthermore investigated if BMSCs and soluble components of the BM microenvironment such as IL-6 and IGF-1, described as major survival and growth factors in MM, would affect the proTAME-induced cell death. Although there was some protection, proTAME was still able to significantly disrupt MM cell survival in the presence of BMSCs and soluble factors. These findings demonstrate that inhibition of the APC/^Cdc20^ would still be active in the BM microenvironment, making it a promising candidate for further preclinical investigation.

As a metaphase arrest is eventually able to trigger cell death, we investigated the activation of apoptotic pathways upon proTAME treatment [6]. ProTAME was able to significantly increase the number of apoptotic cells in different HMCLs. This was accompanied by caspase 3, 8, 9 and PARP cleavage, indicating the involvement of both intrinsic and extrinsic apoptotic pathways. Moreover H2AX was phosphorylated upon proTAME treatment indicating that proTAME treatment could be associated with DNA damage. This confirms previous findings that DNA damage occurs during mitotic arrest with microtubule inhibitors [33, 34]. On the other hand it is described that H2AX can be phosphorylated during cell cycle progression and cell cycle arrest in the absence of DNA damage [35]. Since proTAME is a chemical inhibitor we wanted to exclude non-specific effects of proTAME. Therefore Cdc20 was silenced with siRNA in the HMCL RPMI-8226 and this resulted in a metaphase arrest and induction of apoptosis similar as proTAME suggesting that the observed effect of proTAME is most likely mediated by inhibition of APC/C^Cdc20^. These results are also in line with previous studies in other cancers where knockdown of Cdc20 resulted in a mitotic arrest and cell death [15–17].

A hallmark of cells arrested in mitosis by microtubule disrupting agents is the phosphorylation of Bcl-2 and Bcl-xL, two anti-apoptotic proteins. Phosphorylation of these proteins leads to inactivation of their anti-apoptotic function [36–38]. It is the upregulation of cyclin B1 which is responsible for the phosphorylation of Bcl-xL [39]. In this study, we observed a clear increase in cyclin B1 and phosphorylation of Bcl-2 and Bcl-xL followed by cell death upon proTAME treatment indicating a mitotic arrest. Mcl-1 is believed to be degraded during mitotic arrest and this process requires Cdc20 [38, 40]. Since Cdc20 is blocked with proTAME, this could explain the absence of Mcl-1 degradation in our experiments. Bim, a pro-apoptotic member of the Bcl-2 family was increased upon proTAME treatment. Bim was recently characterized as a substrate of the APC/C^Cdc20^ complex [41]. The apoptotic consequences of depleting Cdc20 have been attributed to the accumulation of Bim. Reducing Bim expression in our cell line significantly reduces the anti-MM effect of proTAME. This might also explain why LP-1 cells which have no Bim expression are the least sensitive to proTAME treatment. Since p53 is a crucial cell cycle and apoptosis regulator we explored p53 protein expression after proTAME treatment. No changes were observed in p53 and phosphorylated p53 (data not shown). Also no link could be observed in the sensitivity of HMCL to proTAME treatment and their p53 status.

To further test whether the APC/C^Cdc20^ complex is a valid target in MM, we combined proTAME with another APC/C inhibitor apcin. Sackton et al. demonstrated that simultaneously blocking the APC/C with proTAME and apcin is synergistic, suggesting that targeting several weak protein-protein interactions may be a good strategy to block large protein complexes such as the APC/C [19]. We confirmed this in our HMCLs as combination treatment with proTAME and apcin significantly induced more apoptosis compared to single treatments. As previously described, apcin alone did not significantly affect MM cell survival probably due to a less efficient inhibition of APC/C compared to proTAME alone. Moreover, we evaluated if proTAME could enhance the anti-MM activity of clinically relevant drugs such as bortezomib and melphalan. ProTAME significantly increased the effect of melphalan. However we did not observe a combination effect with bortezomib. This could be explained by the fact that proteasome inhibition generally induces an interphase arrest which would block the ability of proTAME to induce a metaphase arrest [18, 42].

In MM, vincristine in combination with doxorubicin and dexamethasone, has been used for relapsed patients and as induction therapy [43, 44]. However vincristine as a single agent has demonstrated low efficacy in MM [45]. Vincristine belongs to the microtubule-targeting drugs and disrupt microtubules leading to the activation of the SAC which subsequently blocks the APC/C [46]. However their use is associated with known side effects, such as peripheral neuropathy through disrupting the microtubules in the neurons. Since this mechanism is SAC dependent, the sensitivity to these microtubule-targeting drugs is variable due to variable SAC activity. Therefore, it is hypothesized that targeting mitotic exit downstream of the SAC, by for example blocking Cdc20 would be beneficial [15]. We indeed demonstrate in this study that the APC/C^Cdc20^ could be an attractive and a new therapeutic target, especially in high-risk MM patients. This strategy can influence the proliferation and cell death of tumor cells without affecting the BM microenvironment. Moreover it can be combined with a clinically relevant drug such as melphalan, making it a promising candidate for further preclinical investigation. Since proTAME is not yet optimized for *in vivo* studies, new and more potent small molecules inhibiting the APC/C^Cdc20^ should be developed and *in vivo* validated.

## MATERIALS AND METHODS

### Cell culture

The HMCLs LP-1, RPMI-8226, OPM-2 and NCI-H929 are obtained from the American Type Culture Collection and the U266 and JJN3 were kindly provided by Prof. Dr. I. Van Riet. All the HMCLs and the bone marrow stromal cells (BMSC) were cultured as previously described and regularly tested for mycoplasma contamination [47–50].

### Reagents

The APC/C inhibitor proTAME, IL-6 and IGF-1 were obtained from R & D systems (Oxon, UK). Apcin was provided by Dr. R.W. King (Department of Cell Biology, Harvard Medical School). Bortezomib was obtained from Selleckchem (Munich, Germany) and melphalan was obtained from Sigma-Aldrich (St. Louis MO, USA).

### Western blot analysis

Cells were harvested, lysed, and protein extracts were blotted as previously described [51]. Primary antibodies were used against cyclin B1 (#4138), Skp2 (#4313), caspase-3 (#9665), caspase-8 (#9746), caspase-9 (#9502), PARP (#9542), γH2AX (#5438), Bim (#2933), Mcl-1 (#5453), Bcl-xL (#2764), pBcl-2 (#2827), Cdc20 (#4823), horseradish peroxidase (HRP)-linked anti-mouse (#7076) and -rabbit (#7074) (Cell Signaling, Leiden, the Netherlands) and Bcl-2 (Sc-492), pBcl-xL (Sc-101644), HRP-linked anti-goat (Sc2020)(Santa Cruz, Heidelberg, Germany). β-actin (#4967) (Cell Signaling) was used as a loading control. The pixel densities of proteins were quantified by ImageJ (Wayne Rasband, NIH, USA).

### Cell viability assay

The viability was measured with the CellTiter-Glo Luminiscent Viability assay (Promega, Leiden, The Netherlands) according to manufacturer's instructions. The relative amount of viable cells was expressed as percentage of untreated cells.

### Cell apoptosis assay

Apoptosis was measured with Annexin V-FITC and 7-AAD (BD Biosciences, Franklin Lakes, NJ, USA) followed by flow cytometric analysis (FACS Canto and Diva software, BD Biosciences) according to manufacturer's instructions.

### Microarray data of primary multiple myeloma cells and HMCLs

For the expression of Cdc20 and Cdh1 in HMCLs we used the Affymetrix data of 42 HMCLs from the University hospital of Heidelberg (Germany) and Montpellier (France). These data can be accessed through ArrayExpress database (E-TABM-1088, E-TABM-937 and E-MEXP-2360).

We used 2 independent cohorts of previously untreated MM patients for the association with gene expression-based high-risk [21–23] and survival analysis. The first cohort contains Affymetrix data of 345 MM patients from the University of Arkansas for Medical Science (UAMS, Little Rock, AR) and is termed the TT2-cohort [52]. These MM patients were treated with total therapy 2 [53]. These data can be accessed at the online Gene Expression Omnibus GSE4581. The second cohort consists of Affymetrix data of 206 MM patients from the University hospital of Heidelberg (Germany) and Montpellier (France) and is termed the HM-cohort. MM patients were treated with high dose therapy and autologous stem cell transplantation [21, 28, 54, 55]. These data can be accessed through ArrayExpress database (E-MTAB-372). Affymetrix probe 202870_at and a cut-off of 379.1 was used for Cdc20. For Cdh1 the 209416_at probe was used with a cut-off of 618.8 for the TT-2 cohort and 243 for the HM-cohort.

### The plasma cell labeling index

Multiparameter flow cytometry immunophenotyping was performed as indicated [56]. The cell cycle was assessed using 4, 6-diamidino-2-phenylindole (DAPI) staining (Sigma Aldrich) and plasma cells in the S phase were quantified using incubation with bromodeoxyuridine (BrdU) for 1 hour, and labelling with an anti-BrdU antibody (APC BrdU flow kit, BD Pharmingen, Le Pont De Claix, France).

### Assessment of metaphase arrest

MM cells were treated with proTAME and cytospins (1 × 10^5^ cells/slide) were stained with May-Grünwald Giemsa (respectively VWR International, Leuven, Belgium and Merck, Darmstadt, Germany). Cells in metaphase were counted based on their morphology using a light microscope. For each cytospin, 3 different fields of at least 100 cells were counted.

### Purification of primary human MM cells and peripheral blood mononuclear cells

BM samples were collected from MM patients for diagnosis and research purpose and blood was collected from healthy individuals after written informed consent in accordance with the Declaration of Helsinki. The research is approved by the Ethic Board of UZ Brussel (B.U.N. 143201316382) and the Tumourbank of Lille (CSTMT102). Mononuclear cells were obtained after Ficoll density gradient centrifugation (Nycomed, Lucron Bioproducts, Zurich, Switzerland) and purified as previously described [57]. Primary BMSCs were obtained from healthy subjects, and maintained in DMEM medium (Lonza, Basel, Switzerland) supplemented with 10% fetal calf serum (HyClone, USA), 10% horse serum (Invitrogen, USA), 100 U/mL penicillin/streptomycin (Lonza), and 2 mM L-glutamine (Lonza).

### Lentiviral production and stable transduction

The RPMI-8226shBim and RPMI-shScr were produced as previously described [24].

### Transfection of siRNA

For knockdown of Cdc20 Dicer-Substrate siRNA duplexes were used at a final concentration of 100 nM (HSC.RNAI.N001255.12.1 (duplex 1) and (HSC.RNAI.N001255.12.2 (duplex 2)). The DS NC1 was used as a control. All Dicer-Substrate siRNA duplexes were purchased at IDT (Leuven, Belgium) The RPMI-8226 were transfected using lipofectamine RNAiMax (Invitrogen, Gent, Belgium) according to the procedure provided by the manufacturer.

### Statistical analysis

Gene expression was analyzed by the web tool GenomicScape [58]. Prognostic significance of Cdc20 and Cdh1 genes was calculated using Maxstat R function and Benjamini Hochberg multiple testing correction as previously described [59, 60]. Survival curves were plotted using the Kaplan-Meier method. All these analyses have been done with R.2.10.1 and bioconductor version 2.5. We compared the gene expression levels from patients with high Cdc20 or Cdh1 expression with patients with low Cdc20 or Cdh1 expression and picked up the genes which had significant different expression for Gene set enrichment analysis (GSEA). Gene set enrichment analysis was carried out by computing overlaps with canonical pathways and gene ontology gene sets obtained from the Broad Institute [61]. Graphical and statistical analysis were performed using Graph Prism 5.01 software. For statistical analysis, all experiments were performed at least 3 times as indicated in the Figure legends. The mean and SD of the results are shown in the graphs. The one-sided Mann-Whitney *U*-test, the student *t*-test and the one-way ANOVA with Bonferonni correction were used and *p* values ≤ 0.05 were considered statistically significant.

## SUPPLEMENTARY MATERIALS FIGURES AND TABLES


