# Supplementary material for: Inhibiting the anaphase promoting complex/cyclosome induces a metaphase arrest and cell death in multiple myeloma cells
Source: Oncotarget. 2015 Dec 26;7(4):4062–76. doi: 10.18632/oncotarget.6768 (PMC4826190; doi:10.18632/oncotarget.6768)
Supplement: Supplementary file 1 [file oncotarget-07-4062-s001.pdf]

Inhibiting the anaphase promoting complex/cyclosome induces a metaphase arrest and cell death in multiple myeloma cells

Supplementary Materials

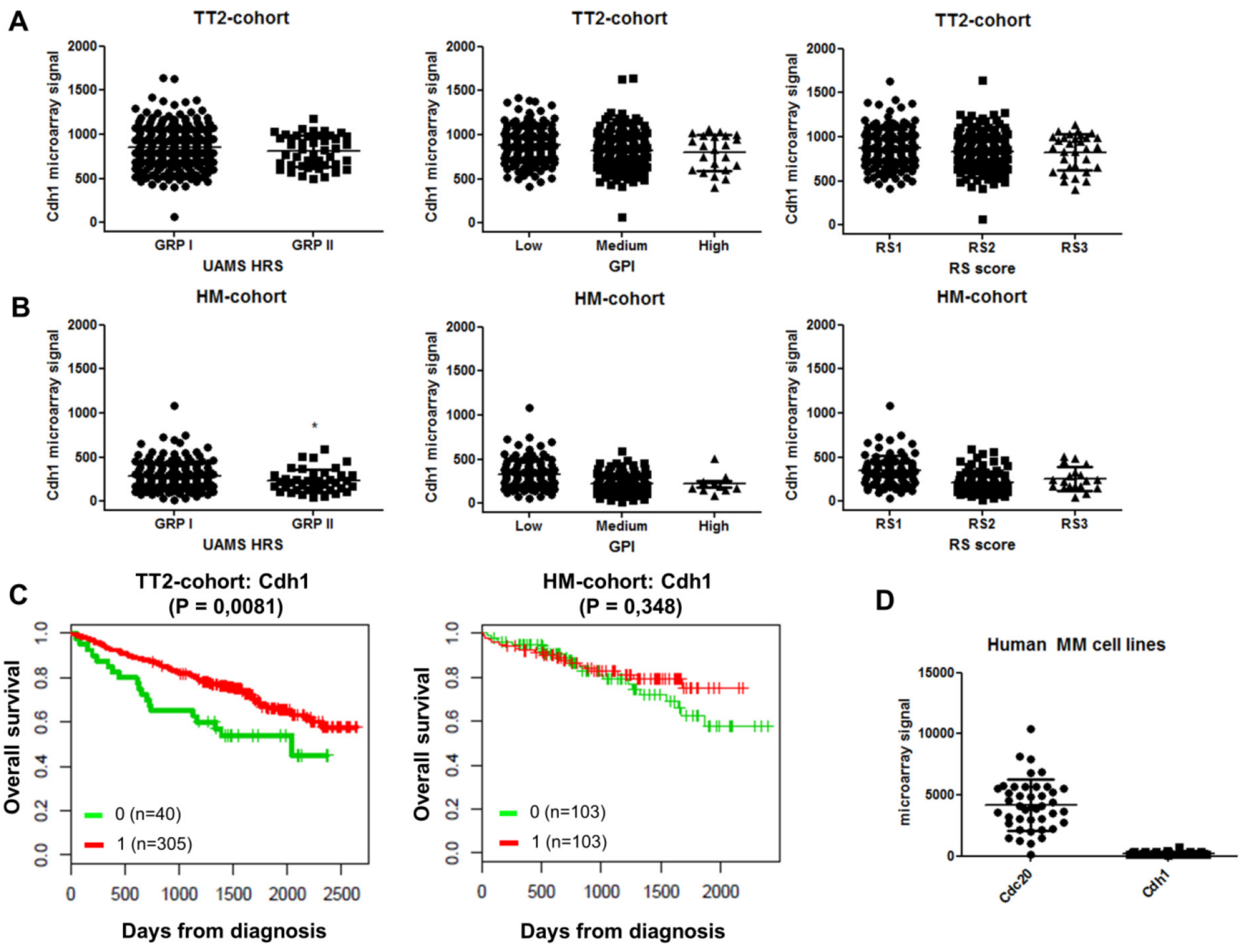

Supplementary Figure S1: Cdh1 expression levels and prognostic value in MM patients. (**A–B**) Association of Cdh1 expression levels in MM patients with gene expression-based high-risk scores in TT2-cohort and HM-cohort. The high-risk groups are compared to the overall mean expression in all groups. * indicates *p*-value is < 0.05 (student *t*-test). (**C**) The overall survival in the TT2-cohort and HM-cohort for low (green) or high (red) Cdh1 expression in MM patients. (**D**) Microarray expression levels of Cdc20 and Cdh1 in different human MM cell lines.

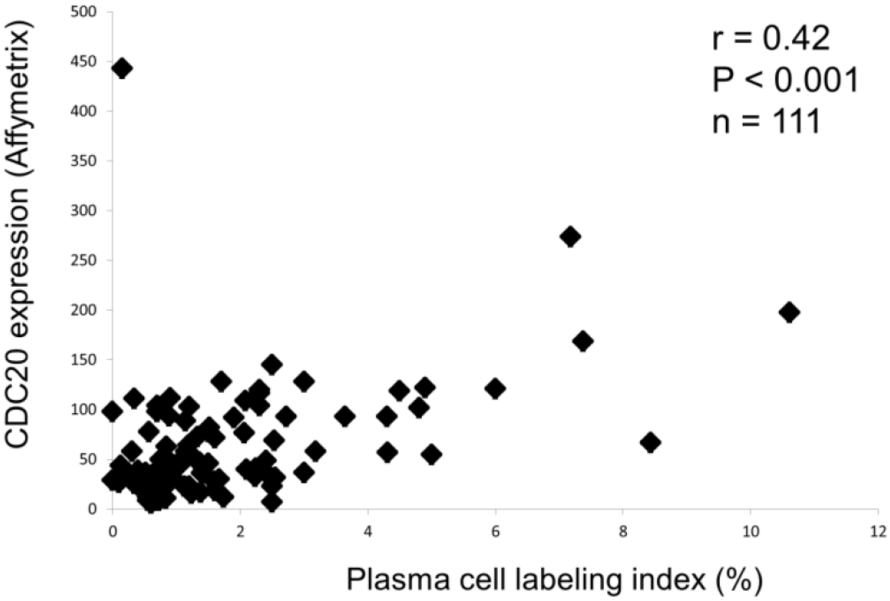

Supplementary Figure S2: Correlation of Cdc20 expression and the plasma cell labeling index. Expression of Cdc20 correlates with the plasma cell labeling index determined by PI staining.

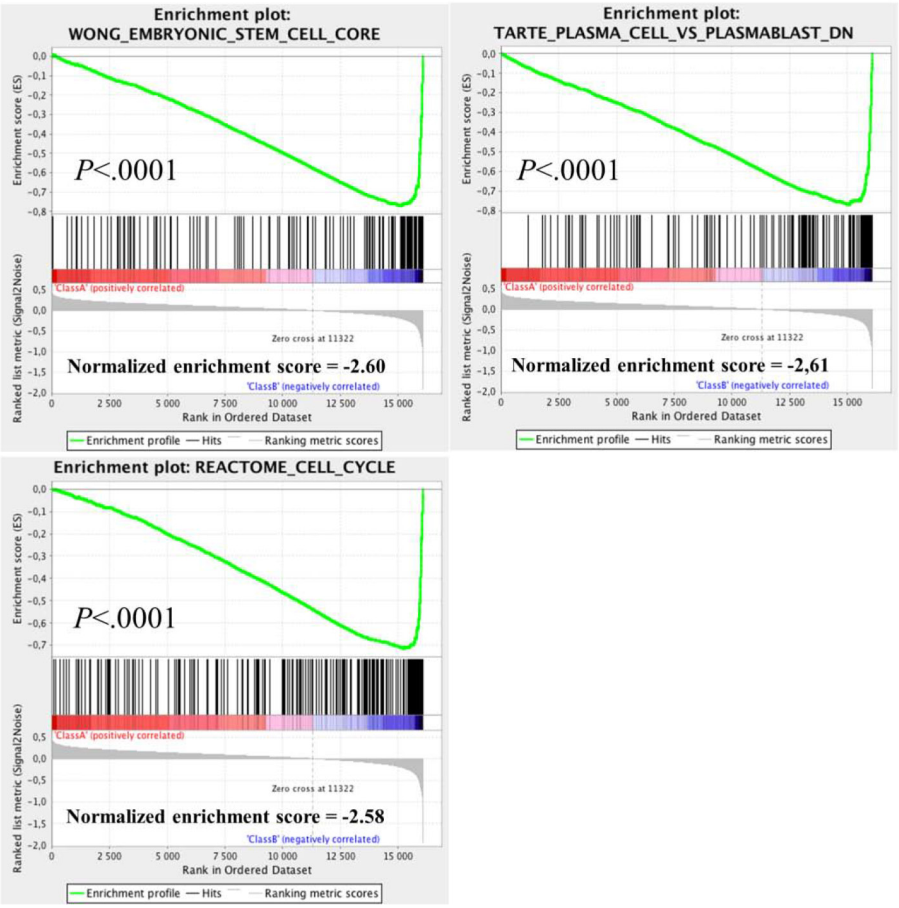

Supplementary Figure S3: Top gene sets significantly associated with high Cdc20 expression in MM. GSEA enrichment plots with the absolute enrichment *p* value and the normalized enrichment score of the gene set.

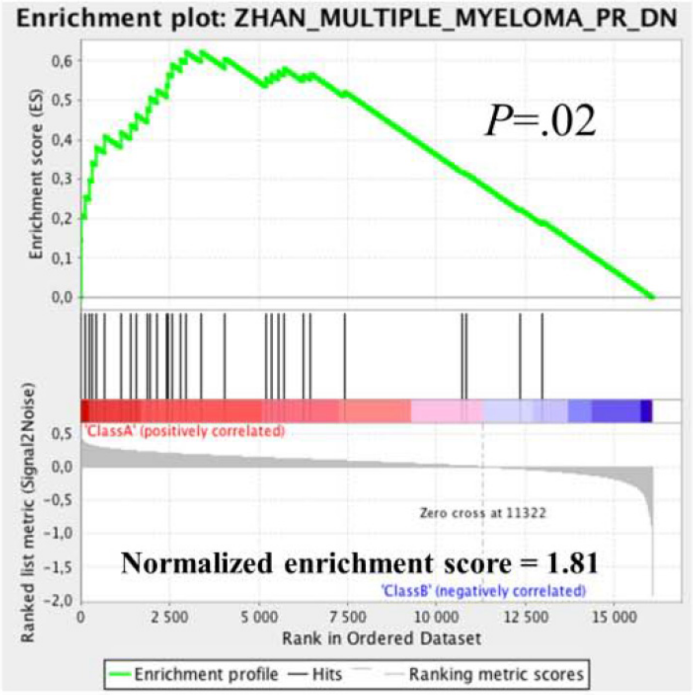

Supplementary Figure S4: Top gene set significantly associated with low Cdc20 expression in MM. GSEA enrichment plot with the absolute enrichment *p* value and the normalized enrichment score of the gene set.

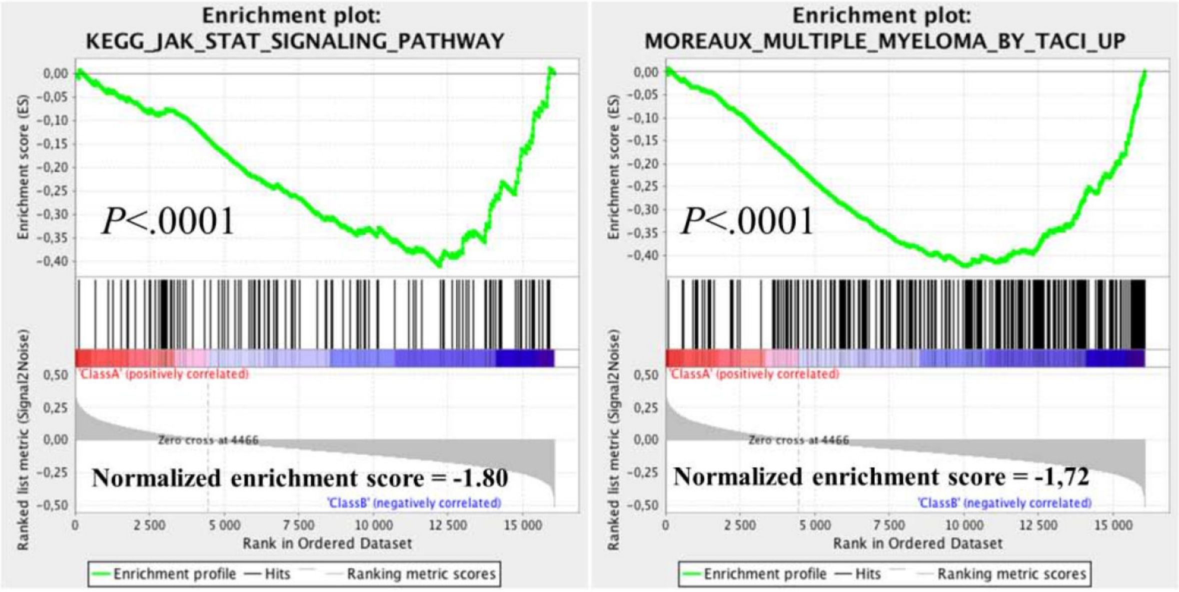

Supplementary Figure S5: Top gene sets significantly associated with high Cdh1 expression in MM. GSEA enrichment plots with the absolute enrichment *p* value and the normalized enrichment score of the gene set.

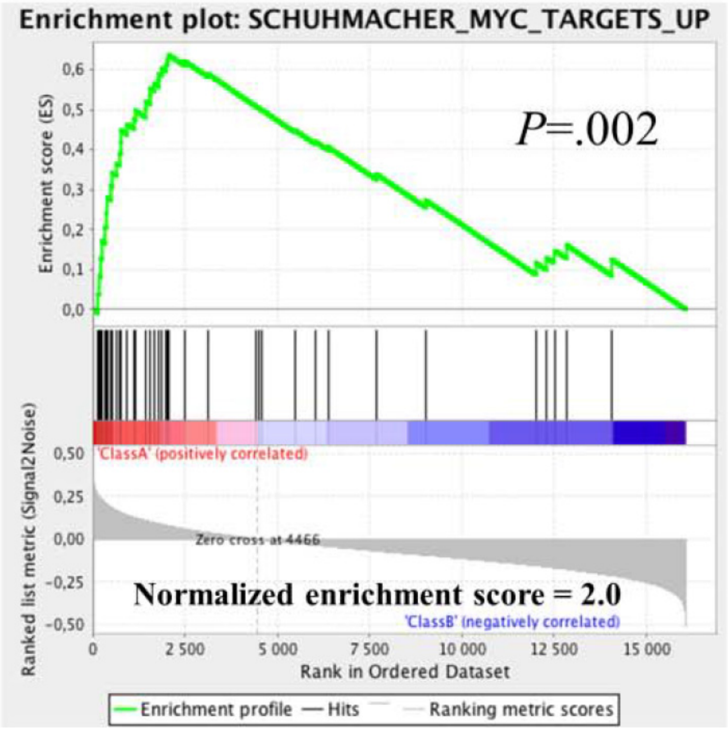

Supplementary Figure S6: Top gene set significantly associated with low Cdh1 expression in MM. GSEA enrichment plot with the absolute enrichment *p* value and the normalized enrichment score of the gene set.

Supplementary Table S1: Genes set enrichment analysis revealed a significant overrepresentation of the gene set WONG_EMBRYONIC_STEM_CELL_CORE in patients with high Cdc20 expression compared to patients with a low expression ($P < 0.0001$)

Probe	Gene Symbol	Gene Title	Rank Metric Score	Running ES	Core Enrichment
MRPS2	MRPS2	mitochondrial ribosomal protein S2	–0,191870853	–0,76920235	Yes
PRPS1	PRPS1	phosphoribosyl pyrophosphate synthetase 1	–0,197177589	–0,76866716	Yes
SUMO1	SUMO1	SMT3 suppressor of mif two 3 homolog 1 (S. cerevisiae)	–0,198594466	–0,7660942	Yes
ALDH7A1	ALDH7A1	aldehyde dehydrogenase 7 family, member A1	–0,19988285	–0,7633119	Yes
WBP11	WBP11	WW domain binding protein 11	–0,200542897	–0,7605192	Yes
PSME3	PSME3	proteasome (prosome, macropain) activator subunit 3 (PA28 gamma; Ki)	–0,208718628	–0,76049435	Yes
GLDC	GLDC	glycine dehydrogenase (decarboxylating)	–0,210937575	–0,7582301	Yes
RCC1	RCC1	regulator of chromosome condensation 1	–0,212119579	–0,7554433	Yes
SERPINH1	SERPINH1	serpin peptidase inhibitor, clade H (heat shock protein 47), member 1, (collagen binding protein 1)	–0,220028609	–0,75398	Yes
ENO1	ENO1	enolase 1, (alpha)	–0,240761608	–0,7564716	Yes
CTSC	CTSC	cathepsin C	–0,246312767	–0,7538369	Yes

DHX9	DHX9	DEAH (Asp-Glu-Ala-His) box polypeptide 9	–0,253485382	–0,75203365	Yes
ELOVL6	ELOVL6	ELOVL family member 6, elongation of long chain fatty acids (FEN1/Elo2, SUR4/Elo3-like, yeast)	–0,265902191	–0,7517345	Yes
MTF2	MTF2	metal response element binding transcription factor 2	–0,268232733	–0,7481235	Yes
NDUFB8	NDUFB8	NADH dehydrogenase (ubiquinone) 1 beta subcomplex, 8, 19kDa	–0,268984258	–0,7441228	Yes
DEK	DEK	DEK oncogene (DNA binding)	–0,280398369	–0,7421459	Yes
MRPL4	MRPL4	mitochondrial ribosomal protein L4	–0,288088083	–0,7395436	Yes
SNX5	SNX5	sorting nexin 5	–0,291674793	–0,7353101	Yes
PPP4C	PPP4C	protein phosphatase 4 (formerly X), catalytic subunit	–0,296679944	–0,73124933	Yes
RUVBL1	RUVBL1	RuvB-like 1 (E. coli)	–0,319730997	–0,73022515	Yes
NIPSNAP1	NIPSNAP1	nipsnap homolog 1 (C. elegans)	–0,323950142	–0,7259222	Yes
NT5DC2	NT5DC2	5’-nucleotidase domain containing 2	–0,329594076	–0,7214041	Yes
CHEK2	CHEK2	CHK2 checkpoint homolog (S. pombe)	–0,342735112	–0,7176859	Yes
PLK1	PLK1	polo-like kinase 1 (Drosophila)	–0,343356729	–0,71225744	Yes
TIMM44	TIMM44	translocase of inner mitochondrial membrane 44 homolog (yeast)	–0,353370219	–0,7079302	Yes
DPP3	DPP3	dipeptidyl-peptidase 3	–0,354383558	–0,7024534	Yes
WDHD1	WDHD1	WD repeat and HMG-box DNA binding protein 1	–0,356864154	–0,69700027	Yes
BAX	BAX	BCL2-associated X protein	–0,366373837	–0,6923415	Yes
NONO	NONO	non-POU domain containing, octamer-binding	–0,367188275	–0,68653625	Yes
RFC3	RFC3	replication factor C (activator 1) 3, 38kDa	–0,367936373	–0,68078214	Yes
CHEK1	CHEK1	CHK1 checkpoint homolog (S. pombe)	–0,369221449	–0,67519665	Yes
POP7	POP7	processing of precursor 7, ribonuclease P subunit (S. cerevisiae)	–0,370075792	–0,6695347	Yes
GTSE1	GTSE1	G-2 and S-phase expressed 1	–0,455218941	–0,667691	Yes
WEE1	WEE1	WEE1 homolog (S. pombe)	–0,456704289	–0,66059643	Yes
CCT5	CCT5	chaperonin containing TCP1, subunit 5 (epsilon)	–0,459631205	–0,65345556	Yes
HN1	HN1	hematological and neurological expressed 1	–0,460738629	–0,6461713	Yes
CKAP2	CKAP2	cytoskeleton associated protein 2	–0,472504288	–0,6391418	Yes
ADSL	ADSL	adenylosuccinate lyase	–0,478549361	–0,6317649	Yes
ANP32E	ANP32E	acidic (leucine-rich) nuclear phosphoprotein 32 family, member E	–0,483442932	–0,6242476	Yes
RPA3	RPA3	replication protein A3, 14kDa	–0,491515487	–0,61666566	Yes

CDC7	CDC7	CDC7 cell division cycle 7 (S. cerevisiae)	–0,497095615	–0,60880655	Yes
MYBL2	MYBL2	v-myb myeloblastosis viral oncogene homolog (avian)-like 2	–0,504296958	–0,6011485	Yes
CSE1L	CSE1L	CSE1 chromosome segregation 1-like (yeast)	–0,510596216	–0,5932019	Yes
CDCA5	CDCA5	cell division cycle associated 5	–0,518205702	–0,58500904	Yes
NASP	NASP	nuclear autoantigenic sperm protein (histone-binding)	–0,522331893	–0,5768769	Yes
HELLS	HELLS	helicase, lymphoid-specific	–0,523693442	–0,56866026	Yes
MCM7	MCM7	MCM7 minichromosome maintenance deficient 7 (S. cerevisiae)	–0,52731055	–0,5603235	Yes
BIRC5	BIRC5	baculoviral IAP repeat-containing 5 (survivin)	–0,529717028	–0,5520116	Yes
CKS2	CKS2	CDC28 protein kinase regulatory subunit 2	–0,530954242	–0,5436172	Yes
MSH2	MSH2	mutS homolog 2, colon cancer, nonpolyposis type 1 (E. coli)	–0,531661868	–0,53533757	Yes
FH	FH	fumarate hydratase	–0,538736999	–0,527072	Yes
PRIM1	PRIM1	primase, polypeptide 1, 49kDa	–0,546554327	–0,5186199	Yes
AURKB	AURKB	aurora kinase B	–0,547838807	–0,50995857	Yes
CHAF1A	CHAF1A	chromatin assembly factor 1, subunit A (p150)	–0,552679837	–0,50122064	Yes
MCM5	MCM5	MCM5 minichromosome maintenance deficient 5, cell division cycle 46 (S. cerevisiae)	–0,563951731	–0,49268243	Yes
CCNF	CCNF	cyclin F	–0,578361809	–0,48379043	Yes
POLE2	POLE2	polymerase (DNA directed), epsilon 2 (p59 subunit)	–0,590895057	–0,47444832	Yes
CCNA2	CCNA2	cyclin A2	–0,5913468	–0,46509913	Yes
DNMT1	DNMT1	DNA (cytosine-5-)-methyltransferase 1	–0,598179996	–0,4558308	Yes
SMC4	SMC4	structural maintenance of chromosomes 4	–0,604619086	–0,4464607	Yes
EXO1	EXO1	exonuclease 1	–0,622215986	–0,43681237	Yes
CDC6	CDC6	CDC6 cell division cycle 6 homolog (S. cerevisiae)	–0,637193918	–0,4271162	Yes
PLK4	PLK4	polo-like kinase 4 (Drosophila)	–0,660085082	–0,41712105	Yes
MCM2	MCM2	MCM2 minichromosome maintenance deficient 2, mitotin (S. cerevisiae)	–0,663495779	–0,40669414	Yes
PCNA	PCNA	proliferating cell nuclear antigen	–0,673896968	–0,39622873	Yes
LMNB1	LMNB1	lamin B1	–0,676305473	–0,3855363	Yes
KIF23	KIF23	kinesin family member 23	–0,684470057	–0,37484077	Yes
TRIP13	TRIP13	thyroid hormone receptor interactor 13	–0,692692518	–0,36420417	Yes
CDCA8	CDCA8	cell division cycle associated 8	–0,701522112	–0,35317606	Yes

GMNN	GMNN	geminin, DNA replication inhibitor	–0,702197433	–0,34207428	Yes
KIF4A	KIF4A	kinesin family member 4A	–0,714693248	–0,3309009	Yes
CKS1B	CKS1B	CDC28 protein kinase regulatory subunit 1B	–0,729257107	–0,3197492	Yes
AURKA	AURKA	aurora kinase A	–0,752391398	–0,3081058	Yes
SPAG5	SPAG5	sperm associated antigen 5	–0,764304757	–0,29652593	Yes
MCM3	MCM3	MCM3 minichromosome maintenance deficient 3 (S. cerevisiae)	–0,767421722	–0,28445593	Yes
NUSAP1	NUSAP1	nucleolar and spindle associated protein 1	–0,767551661	–0,27232093	Yes
TTK	TTK	TTK protein kinase	–0,773475707	–0,26021817	Yes
SMC2	SMC2	structural maintenance of chromosomes 2	–0,776842713	–0,24799925	Yes
CDCA3	CDCA3	cell division cycle associated 3	–0,787610173	–0,23567306	Yes
NCAPH	NCAPH	non-SMC condensin I complex, subunit H	–0,790859818	–0,22316952	Yes
HMGB2	HMGB2	high-mobility group box 2	–0,790866613	–0,21066588	Yes
BUB1B	BUB1B	BUB1 budding uninhibited by benzimidazoles 1 homolog beta (yeast)	–0,797259688	–0,19806117	Yes
RACGAP1	RACGAP1	Rac GTPase activating protein 1	–0,80554533	–0,18532546	Yes
NEK2	NEK2	NIMA (never in mitosis gene a)-related kinase 2	–0,812657177	–0,17260328	Yes
MCM4	MCM4	MCM4 minichromosome maintenance deficient 4 (S. cerevisiae)	–0,85459584	–0,15940697	Yes
KIF11	KIF11	kinesin family member 11	–0,86446619	–0,14599164	Yes
CDKN3	CDKN3	cyclin-dependent kinase inhibitor 3 (CDK2-associated dual specificity phosphatase)	–0,878835261	–0,13234913	Yes
RRM2	RRM2	ribonucleotide reductase M2 polypeptide	–0,882133007	–0,11840257	Yes
BUB1	BUB1	BUB1 budding uninhibited by benzimidazoles 1 homolog (yeast)	–0,887978435	–0,10436359	Yes
CCNB2	CCNB2	cyclin B2	–0,914496243	–0,090031326	Yes
MAD2L1	MAD2L1	MAD2 mitotic arrest deficient-like 1 (yeast)	–0,918614149	–0,07550799	Yes
DTL	DTL	denticleless homolog (Drosophila)	–0,920384467	–0,060956668	Yes
KIF20A	KIF20A	kinesin family member 20A	–0,944989026	–0,04601635	Yes
TOP2A	TOP2A	topoisomerase (DNA) II alpha 170kDa	–0,999249876	–0,030281141	Yes
CDC20	CDC20	CDC20 cell division cycle 20 homolog (S. cerevisiae)	–1,915308833	–1,92E–08	Yes

Supplementary Table S2: Genes set enrichment analysis revealed a significant overrepresentation of the gene set TARTE_PLASMA_CELL_VS_PLASMABLAST_DN in patients with high Cdc20 expression compared to patients with a low expression ($P < 0.0001$)

Probe	Gene Symbol	Gene Titel	Rank Metric Score	Running ES	Core Enrichment
NNT	NNT	nicotinamide nucleotide transhydrogenase	–0,185168803	–0,7686341	Yes
EIF5	EIF5	eukaryotic translation initiation factor 5	–0,189564899	–0,76693934	Yes
TOPBP1	TOPBP1	topoisomerase (DNA) II binding protein 1	–0,191578746	–0,7646409	Yes
GIT2	GIT2	G protein-coupled receptor kinase interactor 2	–0,194381312	–0,76210237	Yes
CLEC2B	CLEC2B	C-type lectin domain family 2, member B	–0,195314094	–0,7593578	Yes
CASP6	CASP6	caspase 6, apoptosis-related cysteine peptidase	–0,197693035	–0,75675887	Yes
SUMO1	SUMO1	SMT3 suppressor of mif two 3 homolog 1 (S. cerevisiae)	–0,198594466	–0,7534505	Yes
IQGAP2	IQGAP2	IQ motif containing GTPase activating protein 2	–0,208418593	–0,75361663	Yes
GLDC	GLDC	glycine dehydrogenase (decarboxylating)	–0,210937575	–0,751028	Yes
PSMB8	PSMB8	proteasome (prosome, macropain) subunit, beta type, 8 (large multifunctional peptidase 7)	–0,218251437	–0,74887294	Yes
EXOSC2	EXOSC2	exosome component 2	–0,222844154	–0,74650806	Yes
IDH2	IDH2	isocitrate dehydrogenase 2 (NADP+), mitochondrial	–0,24610506	–0,7485694	Yes
CORO1A	CORO1A	coronin, actin binding protein, 1A	–0,249765813	–0,7450834	Yes
LILRB4	LILRB4	leukocyte immunoglobulin-like receptor, subfamily B (with TM and ITIM domains), member 4	–0,264668196	–0,74466425	Yes
BCL3	BCL3	B-cell CLL/lymphoma 3	–0,283899784	–0,74458724	Yes
ASNS	ASNS	asparagine synthetase	–0,284011483	–0,73946846	Yes
PPP4C	PPP4C	protein phosphatase 4 (formerly X), catalytic subunit	–0,296679944	–0,7361344	Yes
TFRC	TFRC	transferrin receptor (p90, CD71)	–0,297989845	–0,7312015	Yes
PPIF	PPIF	peptidylprolyl isomerase F (cyclophilin F)	–0,300260097	–0,7261643	Yes
ATP13A3	ATP13A3	ATPase type 13A3	–0,301329613	–0,72079253	Yes
MAPKAPK3	MAPKAPK3	mitogen-activated protein kinase-activated protein kinase 3	–0,316597015	–0,7172841	Yes
WARS	WARS	tryptophanyl-tRNA synthetase	–0,319976807	–0,71201307	Yes

SLC19A1	SLC19A1	solute carrier family 19 (folate transporter), member 1	–0,320311278	–0,706232	Yes
POLD2	POLD2	polymerase (DNA directed), delta 2, regulatory subunit 50kDa	–0,34615773	–0,7033812	Yes
SNRPF	SNRPF	small nuclear ribonucleoprotein polypeptide F	–0,348027378	–0,6972834	Yes
IFI30	IFI30	interferon, gamma-inducible protein 30	–0,349886477	–0,69108874	Yes
UCK2	UCK2	uridine-cytidine kinase 2	–0,358571261	–0,6855545	Yes
ATP1B3	ATP1B3	ATPase, Na+/K+ transporting, beta 3 polypeptide	–0,361074358	–0,6792817	Yes
SMARCA4	SMARCA4	SWI/SNF related, matrix associated, actin dependent regulator of chromatin, subfamily a, member 4	–0,361651272	–0,6727464	Yes
RFC3	RFC3	replication factor C (activator 1) 3, 38kDa	–0,367936373	–0,66666335	Yes
CDC25B	CDC25B	cell division cycle 25B	–0,37087211	–0,6605268	Yes
HPRT1	HPRT1	hypoxanthine phosphoribosyltransferase 1 (Lesch-Nyhan syndrome)	–0,372962803	–0,65397406	Yes
LBR	LBR	lamin B receptor	–0,373637617	–0,6472201	Yes
SLC7A5	SLC7A5	solute carrier family 7 (cationic amino acid transporter, y+ system), member 5	–0,374481559	–0,64038765	Yes
PSMD4	PSMD4	proteasome (prosome, macropain) 26S subunit, non-ATPase, 4	–0,381905943	–0,6341128	Yes
TMEM97	TMEM97	transmembrane protein 97	–0,38246125	–0,62719774	Yes
CBX5	CBX5	chromobox homolog 5 (HP1 alpha homolog, Drosophila)	–0,387424916	–0,6203182	Yes
LGALS1	LGALS1	lectin, galactoside-binding, soluble, 1 (galectin 1)	–0,404439211	–0,61407316	Yes
ACOT7	ACOT7	acyl-CoA thioesterase 7	–0,445956647	–0,60788953	Yes
PSMC3IP	PSMC3IP	PSMC3 interacting protein	–0,454770476	–0,5999073	Yes
CCT5	CCT5	chaperonin containing TCP1, subunit 5 (epsilon)	–0,459631205	–0,5918993	Yes
MTHFD1	MTHFD1	methylenetetrahydrofolate dehydrogenase (NADP+ dependent) 1, methenyltetrahydrofolate cyclohydrolase, formyltetrahydrofolate synthetase	–0,477121979	–0,58388716	Yes
CCT2	CCT2	chaperonin containing TCP1, subunit 2 (beta)	–0,497349858	–0,575443	Yes

CSE1L	CSE1L	CSE1 chromosome segregation 1-like (yeast)	–0,510596216	–0,5665052	Yes
NASP	NASP	nuclear autoantigenic sperm protein (histone-binding)	–0,522331893	–0,55716425	Yes
KIFC1	KIFC1	kinesin family member C1	–0,52843678	–0,5477119	Yes
CKS2	CKS2	CDC28 protein kinase regulatory subunit 2	–0,530954242	–0,53808767	Yes
TROAP	TROAP	trophinin associated protein (tastin)	–0,531657934	–0,52845055	Yes
FH	FH	fumarate hydratase	–0,538736999	–0,51893634	Yes
PRIM1	PRIM1	primase, polypeptide 1, 49kDa	–0,546554327	–0,5091534	Yes
CHAF1A	CHAF1A	chromatin assembly factor 1, subunit A (p150)	–0,552679837	–0,4991328	Yes
CTPS	CTPS	CTP synthase	–0,553112745	–0,48910427	Yes
CCNF	CCNF	cyclin F	–0,578361809	–0,47911906	Yes
CCNA2	CCNA2	cyclin A2	–0,5913468	–0,46839294	Yes
FOXM1	FOXM1	forkhead box M1	–0,617652893	–0,45775384	Yes
ESPL1	ESPL1	extra spindle poles like 1 (S. cerevisiae)	–0,633353293	–0,44645032	Yes
CDC6	CDC6	CDC6 cell division cycle 6 homolog (S. cerevisiae)	–0,637193918	–0,43495074	Yes
GINS1	GINS1	GINS complex subunit 1 (Psf1 homolog)	–0,648120284	–0,42325178	Yes
TMPO	TMPO	thymopoietin	–0,651171029	–0,4113712	Yes
PLK4	PLK4	polo-like kinase 4 (Drosophila)	–0,660085082	–0,39951694	Yes
HMMR	HMMR	hyaluronan-mediated motility receptor (RHAMM)	–0,673276722	–0,38748503	Yes
PCNA	PCNA	proliferating cell nuclear antigen	–0,673896968	–0,3751898	Yes
LMNB1	LMNB1	lamin B1	–0,676305473	–0,36285064	Yes
CENPA	CENPA	centromere protein A	–0,680396974	–0,3504998	Yes
KIF23	KIF23	kinesin family member 23	–0,684470057	–0,33801168	Yes
KIAA0101	KIAA0101	KIAA0101	–0,68695128	–0,32554126	Yes
TRIP13	TRIP13	thyroid hormone receptor interactor 13	–0,692692518	–0,31309208	Yes
RFC4	RFC4	replication factor C (activator 1) 4, 37kDa	–0,723059058	–0,30040386	Yes
CKS1B	CKS1B	CDC28 protein kinase regulatory subunit 1B	–0,729257107	–0,2872876	Yes
KIF14	KIF14	kinesin family member 14	–0,753028214	–0,2739266	Yes
CENPE	CENPE	centromere protein E, 312kDa	–0,75834018	–0,2601537	Yes
TYMS	TYMS	thymidylate synthetase	–0,762543559	–0,24636711	Yes
EZH2	EZH2	enhancer of zeste homolog 2 (Drosophila)	–0,762817562	–0,23244953	Yes

MCM3	MCM3	MCM3 minichromosome maintenance deficient 3 (S. cerevisiae)	–0,767421722	–0,21857393	Yes
UBE2C	UBE2C	ubiquitin-conjugating enzyme E2C	–0,772211015	–0,20461096	Yes
TTK	TTK	TTK protein kinase	–0,773475707	–0,19049892	Yes
HMGB2	HMGB2	high-mobility group box 2	–0,790866613	–0,17644757	Yes
CCNB1	CCNB1	cyclin B1	–0,810844064	–0,16184273	Yes
NEK2	NEK2	NIMA (never in mitosis gene a)-related kinase 2	–0,812657177	–0,14701582	Yes
MKI67	MKI67	antigen identified by monoclonal antibody Ki-67	–0,815378487	–0,13213927	Yes
GGH	GGH	gamma-glutamyl hydrolase (conjugase, folylpolygammaglutamyl hydrolase)	–0,849506497	–0,11676604	Yes
KIF11	KIF11	kinesin family member 11	–0,86446619	–0,10137186	Yes
CDKN3	CDKN3	cyclin-dependent kinase inhibitor 3 (CDK2-associated dual specificity phosphatase)	–0,878835261	–0,08558953	Yes
RRM2	RRM2	ribonucleotide reductase M2 polypeptide	–0,882133007	–0,069495045	Yes
MAD2L1	MAD2L1	MAD2 mitotic arrest deficient-like 1 (yeast)	–0,918614149	–0,052986942	Yes
TOP2A	TOP2A	topoisomerase (DNA) II alpha 170kDa	–0,999249876	–0,034944646	Yes
CDC20	CDC20	CDC20 cell division cycle 20 homolog (S. cerevisiae)	-1,915308833	9,95E–08	Yes

Supplementary Table S3: Genes set enrichment analysis revealed a significant overrepresentation of the gene set REACTOME_CELL_CYCLE in patients with high Cdc20 expression compared to patients with a low expression (*P* < 0.0001)

Probe	Gene Symbol	Gene Title	Rank Metric Score	Running ES	Core Enrichment
CCNE2	CCNE2	cyclin E2	–0,210470393	–0,7122567	Yes
CETN2	CETN2	centrin, EF-hand protein, 2	–0,215965241	–0,7107932	Yes
PSMB8	PSMB8	proteasome (prosome, macropain) subunit, beta type, 8 (large multifunctional peptidase 7)	–0,218251437	–0,70815873	Yes
TUBGCP3	TUBGCP3	tubulin, gamma complex associated protein 3	–0,227337554	–0,707796	Yes
ACTR1A	ACTR1A	ARP1 actin-related protein 1 homolog A, centractin alpha (yeast)	–0,244802833	–0,70907867	Yes
DKC1	DKC1	dyskeratosis congenita 1, dyskerin	–0,256263882	–0,7093113	Yes
CDKN2A	CDKN2A	cyclin-dependent kinase inhibitor 2A (melanoma, p16, inhibits CDK4)	–0,264931947	–0,7079019	Yes
RBL2	RBL2	retinoblastoma-like 2 (p130)	–0,266335845	–0,70470196	Yes
CDK6	CDK6	cyclin-dependent kinase 6	–0,267403454	–0,7010442	Yes
TFDP1	TFDP1	transcription factor Dp-1	–0,27324456	–0,6986933	Yes
CDT1	CDT1	chromatin licensing and DNA replication factor 1	–0,28196615	–0,69634324	Yes
H2AFX	H2AFX	H2A histone family, member X	–0,289916635	–0,6941318	Yes
CENPJ	CENPJ	centromere protein J	–0,313174903	–0,693672	Yes
RUVBL1	RUVBL1	RuvB-like 1 (E. coli)	–0,319730997	–0,68983036	Yes
CENPL	CENPL	centromere protein L	–0,322648495	–0,685757	Yes
RANGAP1	RANGAP1	Ran GTPase activating protein 1	–0,337927371	–0,68291795	Yes
CHEK2	CHEK2	CHK2 checkpoint homolog (S. pombe)	–0,342735112	–0,6786188	Yes
PLK1	PLK1	polo-like kinase 1 (Drosophila)	–0,343356729	–0,6736785	Yes
POLD2	POLD2	polymerase (DNA directed), delta 2, regulatory subunit 50kDa	–0,34615773	–0,6689508	Yes
RFC3	RFC3	replication factor C (activator 1) 3, 38kDa	–0,367936373	–0,66643864	Yes
CHEK1	CHEK1	CHK1 checkpoint homolog (S. pombe)	–0,369221449	–0,6613791	Yes
CDC25B	CDC25B	cell division cycle 25B	–0,37087211	–0,656359	Yes
E2F1	E2F1	E2F transcription factor 1	–0,372754276	–0,6511854	Yes

MCM8	MCM8	MCM8 minichromosome maintenance deficient 8 (S. cerevisiae)	–0,375439107	–0,64622605	Yes
APITD1	APITD1	apoptosis-inducing, TAF9-like domain 1	–0,376720846	–0,6409953	Yes
RFC5	RFC5	replication factor C (activator 1) 5, 36.5kDa	–0,37741527	–0,6356282	Yes
PSMD4	PSMD4	proteasome (prosome, macropain) 26S subunit, non-ATPase, 4	–0,381905943	–0,63019645	Yes
RFC2	RFC2	replication factor C (activator 1) 2, 40kDa	–0,395735234	–0,6255141	Yes
CENPO	CENPO	centromere protein O	–0,407026917	–0,6202267	Yes
CDKN2C	CDKN2C	cyclin-dependent kinase inhibitor 2C (p18, inhibits CDK4)	–0,441182077	–0,61545944	Yes
WEE1	WEE1	WEE1 homolog (S. pombe)	–0,456704289	–0,60971016	Yes
KIF18A	KIF18A	kinesin family member 18A	–0,466508269	–0,60331404	Yes
CENPM	CENPM	centromere protein M	–0,467451692	–0,5965882	Yes
HIST1H4C	HIST1H4C	histone cluster 1, H4c	–0,469853073	–0,5898911	Yes
LIG1	LIG1	ligase I, DNA, ATP-dependent	–0,476820141	–0,5832201	Yes
RFWD2	RFWD2	ring finger and WD repeat domain 2	–0,488050491	–0,5766405	Yes
RPA3	RPA3	replication protein A3, 14kDa	–0,491515487	–0,56963164	Yes
CDC7	CDC7	CDC7 cell division cycle 7 (S. cerevisiae)	–0,497095615	–0,5624793	Yes
MYBL2	MYBL2	v-myb myeloblastosis viral oncogene homolog (avian)-like 2	–0,504296958	–0,5555395	Yes
MCM7	MCM7	MCM7 minichromosome maintenance deficient 7 (S. cerevisiae)	–0,52731055	–0,5485214	Yes
BIRC5	BIRC5	baculoviral IAP repeat-containing 5 (survivin)	–0,529717028	–0,54096293	Yes
GINS2	GINS2	GINS complex subunit 2 (Psf2 homolog)	–0,533452272	–0,53354037	Yes
CASC5	CASC5	cancer susceptibility candidate 5	–0,54503876	–0,52607757	Yes
PRIM1	PRIM1	primase, polypeptide 1, 49kDa	–0,546554327	–0,51821357	Yes
AURKB	AURKB	aurora kinase B	–0,547838807	–0,51033115	Yes
MCM6	MCM6	MCM6 minichromosome maintenance deficient 6 (MIS5 homolog, S. pombe) (S. cerevisiae)	–0,555930495	–0,50252193	Yes

MCM5	MCM5	MCM5 minichromosome maintenance deficient 5, cell division cycle 46 (S. cerevisiae)	–0,563951731	–0,49459732	Yes
POLA1	POLA1	polymerase (DNA directed), alpha 1	–0,574716508	–0,48651782	Yes
POLE2	POLE2	polymerase (DNA directed), epsilon 2 (p59 subunit)	–0,590895057	–0,47807908	Yes
CCNA2	CCNA2	cyclin A2	–0,5913468	–0,46957064	Yes
CENPH	CENPH	centromere protein H	–0,597544909	–0,46109945	Yes
OIP5	OIP5	Opa interacting protein 5	–0,60263294	–0,45255506	Yes
FEN1	FEN1	flap structure-specific endonuclease 1	–0,602996886	–0,44387898	Yes
HSP90AA1	HSP90AA1	heat shock protein 90kDa alpha (cytosolic), class A member 1	–0,605882704	–0,4352246	Yes
ZWILCH	ZWILCH	Zwilch, kinetochore associated, homolog (Drosophila)	–0,63061744	–0,42646724	Yes
CDC6	CDC6	CDC6 cell division cycle 6 homolog (S. cerevisiae)	–0,637193918	–0,41748878	Yes
GINS1	GINS1	GINS complex subunit 1 (Psf1 homolog)	–0,648120284	–0,4082899	Yes
CDC25A	CDC25A	cell division cycle 25A	–0,651812792	–0,3989747	Yes
PLK4	PLK4	polo-like kinase 4 (Drosophila)	–0,660085082	–0,38960364	Yes
MCM2	MCM2	MCM2 minichromosome maintenance deficient 2, mitotin (S. cerevisiae)	–0,663495779	–0,38012034	Yes
CENPK	CENPK	centromere protein K	–0,665057182	–0,37055132	Yes
FBXO5	FBXO5	F-box protein 5	–0,670446992	–0,36090475	Yes
PCNA	PCNA	proliferating cell nuclear antigen	–0,673896968	–0,35127175	Yes
LMNB1	LMNB1	lamin B1	–0,676305473	–0,3415409	Yes
CENPA	CENPA	centromere protein A	–0,680396974	–0,3318144	Yes
KIF23	KIF23	kinesin family member 23	–0,684470057	–0,32196605	Yes
MCM10	MCM10	MCM10 minichromosome maintenance deficient 10 (S. cerevisiae)	–0,685005903	–0,31211	Yes
CENPN	CENPN	centromere protein N	–0,688516378	–0,3022667	Yes
CDCA8	CDCA8	cell division cycle associated 8	–0,701522112	–0,2924259	Yes
GMNN	GMNN	geminin, DNA replication inhibitor	–0,702197433	–0,2823225	Yes
BRCA1	BRCA1	breast cancer 1, early onset	–0,712645292	–0,272132	Yes
MLF1IP	MLF1IP	MLF1 interacting protein	–0,721346676	–0,26187953	Yes
RFC4	RFC4	replication factor C (activator 1) 4, 37kDa	–0,723059058	–0,25147596	Yes

CKS1B	CKS1B	CDC28 protein kinase regulatory subunit 1B	–0,729257107	–0,2411729	Yes
E2F2	E2F2	E2F transcription factor 2	–0,748274207	–0,23053297	Yes
AURKA	AURKA	aurora kinase A	–0,752391398	–0,2197706	Yes
TYMS	TYMS	thymidylate synthetase	–0,762543559	–0,20917825	Yes
MCM3	MCM3	MCM3 minichromosome maintenance deficient 3 (S. cerevisiae)	–0,767421722	–0,19832607	Yes
UBE2C	UBE2C	ubiquitin-conjugating enzyme E2C	–0,772211015	–0,18734173	Yes
PTTG1	PTTG1	pituitary tumor-transforming 1	–0,77743268	–0,1763455	Yes
BUB1B	BUB1B	BUB1 budding uninhibited by benzimidazoles 1 homolog beta (yeast)	–0,797259688	–0,16512722	Yes
CCNB1	CCNB1	cyclin B1	–0,810844064	–0,15358703	Yes
NEK2	NEK2	NIMA (never in mitosis gene a)-related kinase 2	–0,812657177	–0,1418943	Yes
MCM4	MCM4	MCM4 minichromosome maintenance deficient 4 (S. cerevisiae)	–0,85459584	–0,12991427	Yes
SGOL2	SGOL2	shugoshin-like 2 (S. pombe)	–0,856163681	–0,11759556	Yes
ZWINT	ZWINT	ZW10 interactor	–0,870476186	–0,105450265	Yes
RRM2	RRM2	ribonucleotide reductase M2 polypeptide	–0,882133007	–0,09288435	Yes
BUB1	BUB1	BUB1 budding uninhibited by benzimidazoles 1 homolog (yeast)	–0,887978435	–0,08010788	Yes
H2AFZ	H2AFZ	H2A histone family, member Z	–0,891756773	–0,06727706	Yes
CCNB2	CCNB2	cyclin B2	–0,914496243	–0,05418227	Yes
MAD2L1	MAD2L1	MAD2 mitotic arrest deficient-like 1 (yeast)	–0,918614149	–0,04096501	Yes
KIF20A	KIF20A	kinesin family member 20A	–0,944989026	–0,027431484	Yes
CDC20	CDC20	CDC20 cell division cycle 20 homolog (S. cerevisiae)	–1,915308833	3,05E–08	Yes

Supplementary Table S4: Genes set enrichment analysis revealed a significant overrepresentation of the gene set ZHAN_MULTIPLE_MYELOMA_PR_DN in patients with low Cdc20 expression compared to patients with a high expression (*P* = 0.02)

Probe	Gene Symbol	Gene Title	Rank Metric Score	Running ES	Core enrichment
SLC7A7	SLC7A7	solute carrier family 7 (cationic amino acid transporter, y+ system), member 7	0,491621882	0,07380156	Yes
BCL10	BCL10	B-cell CLL/lymphoma 10	0,470807523	0,14489654	Yes
ZBTB20	ZBTB20	zinc finger and BTB domain containing 20	0,427429378	0,20844337	Yes
INADL	INADL	InaD-like (Drosophila)	0,355239719	0,2552273	Yes
NR3C2	NR3C2	nuclear receptor subfamily 3, group C, member 2	0,33040756	0,29832378	Yes
RASGRP3	RASGRP3	RAS guanyl releasing protein 3 (calcium and DAG-regulated)	0,317401618	0,34120232	Yes
PLCL1	PLCL1	phospholipase C-like 1	0,306086928	0,3815616	Yes
FGF2	FGF2	fibroblast growth factor 2 (basic)	0,283091187	0,40890735	Yes
SYNE2	SYNE2	spectrin repeat containing, nuclear envelope 2	0,254032373	0,41914368	Yes
ALCAM	ALCAM	activated leukocyte cell adhesion molecule	0,240861133	0,4387405	Yes
DPYD	DPYD	dihydropyrimidine dehydrogenase	0,233723566	0,4634955	Yes
TFEB	TFEB	transcription factor EB	0,221070349	0,47817063	Yes
ARID5B	ARID5B	AT rich interactive domain 5B (MRF1-like)	0,218854621	0,50679153	Yes
SOCS1	SOCS1	suppressor of cytokine signaling 1	0,21193935	0,5255755	Yes
JMJD1C	JMJD1C	jumonji domain containing 1C	0,202201977	0,53902274	Yes
BLNK	BLNK	B-cell linker	0,200587749	0,56619483	Yes
DUSP26	DUSP26	dual specificity phosphatase 26 (putative)	0,197888374	0,5909637	Yes
FGD4	FGD4	FYVE, RhoGEF and PH domain containing 4	0,190971345	0,60340106	Yes
ANKRD44	ANKRD44	ankyrin repeat domain 44	0,187772036	0,62396085	Yes
PHF1	PHF1	PHD finger protein 1	0,177340522	0,62392575	No
ZHX2	ZHX2	zinc fingers and homeoboxes 2	0,160531506	0,6062614	No
PRDM2	PRDM2	PR domain containing 2, with ZNF domain	0,134613976	0,55462587	No

SATB1	SATB1	special AT-rich sequence binding protein 1 (binds to nuclear matrix/scaffold-associating DNA's)	0,131713346	0,5650367	No
CYLD	CYLD	cylindromatosis (turban tumor syndrome)	0,12784791	0,5728684	No
MAP2	MAP2	microtubule-associated protein 2	0,124305077	0,5808511	No
CNTN1	CNTN1	contactin 1	0,113127686	0,5645716	No
EPS15	EPS15	epidermal growth factor receptor pathway substrate 15	0,108968459	0,56805575	No
GAB1	GAB1	GRB2-associated binding protein 1	0,0896056	0,52284396	No
ARHGAP18	ARHGAP18	Rho GTPase activating protein 18	0,016339948	0,3189008	No
RSBN1	RSBN1	round spermatid basic protein 1	0,013282404	0,31361043	No
TK2	TK2	thymidine kinase 2, mitochondrial	–0,030378854	0,22434652	No
PPAPDC1B	PPAPDC1B	phosphatidic acid phosphatase type 2 domain containing 1B	–0,052189939	0,193128	No

Supplementary Table S5: Genes set enrichment analysis revealed a significant overrepresentation of the gene set MOREAUX_MULTIPLE_MYELOMA_BY_TACI_UP in patients with high Cdh1 expression compared to patients with a low expression (*P* < 0.0001)

Probe	Gene Symbol	Gene Title	Rank Metric Score	Running ES	Core Enrichment
PCDH1	PCDH1	protocadherin 1 (cadherin-like 1)	–0,115214884	–0,4217955	Yes
VGLL3	VGLL3	vestigial like 3 (Drosophila)	–0,115244761	–0,4192074	Yes
MAPK8IP2	MAPK8IP2	mitogen-activated protein kinase 8 interacting protein 2	–0,115508609	–0,4175001	Yes
PMP2	PMP2	peripheral myelin protein 2	–0,116589256	–0,4176672	Yes
EEF1A2	EEF1A2	eukaryotic translation elongation factor 1 alpha 2	–0,117680736	–0,4184411	Yes
PHF1	PHF1	PHD finger protein 1	–0,117765352	–0,4157275	Yes
PVRL2	PVRL2	poliovirus receptor-related 2 (herpesvirus entry mediator B)	–0,117789462	–0,4130133	Yes
PCDH11X	PCDH11X	protocadherin 11 X-linked	–0,118301734	–0,4115539	Yes
ACSL6	ACSL6	acyl-CoA synthetase long-chain family member 6	–0,118335292	–0,4086361	Yes
PHLDA1	PHLDA1	pleckstrin homology-like domain, family A, member 1	–0,119399719	–0,4088607	Yes
KCNK10	KCNK10	potassium channel, subfamily K, member 10	–0,121714652	–0,4126403	Yes
AKR1C1	AKR1C1	aldo-keto reductase family 1, member C1 (dihydrodiol dehydrogenase 1; 20-alpha (3-alpha)-hydroxysteroid dehydrogenase)	–0,122373074	–0,4115875	Yes
C3AR1	C3AR1	complement component 3a receptor 1	–0,123345174	–0,411588	Yes
CASZ1	CASZ1	castor homolog 1, zinc finger (Drosophila)	–0,123588949	–0,4093012	Yes
NF1	NF1	neurofibromin 1 (neurofibromatosis, von Recklinghausen disease, Watson disease)	–0,123954535	–0,4074489	Yes
REL	REL	v-rel reticuloendotheliosis viral oncogene homolog (avian)	–0,12426503	–0,4055256	Yes
SLC4A10	SLC4A10	solute carrier family 4, sodium bicarbonate transporter-like, member 10	–0,124285921	–0,4024611	Yes
STYK1	STYK1	serine/threonine/tyrosine kinase 1	–0,124629006	–0,4001486	Yes
RRBP1	RRBP1	ribosome binding protein 1 homolog 180kDa (dog)	–0,127582863	–0,4052411	Yes
CXCL1	CXCL1	chemokine (C-X-C motif) ligand 1 (melanoma growth stimulating activity, alpha)	–0,127637669	–0,4021573	Yes
PLA2G5	PLA2G5	phospholipase A2, group V	–0,130423516	–0,4073699	Yes
HORMAD2	HORMAD2	HORMA domain containing 2	–0,131945342	–0,4083624	Yes

HUS1	HUS1	HUS1 checkpoint homolog (S. pombe)	–0,134599045	–0,4135354	Yes
FXYD3	FXYD3	FXYD domain containing ion transport regulator 3	–0,135771438	–0,4125958	Yes
EPB41L1	EPB41L1	erythrocyte membrane protein band 4.1-like 1	–0,13634859	–0,4108182	Yes
TNFRSF13B	TNFRSF13B	tumor necrosis factor receptor superfamily, member 13B	–0,137477383	–0,411611	Yes
OR2C1	OR2C1	olfactory receptor, family 2, subfamily C, member 1	–0,137672603	–0,4088501	Yes
PTCRA	PTCRA	pre T-cell antigen receptor alpha	–0,138061747	–0,4067767	Yes
S100A12	S100A12	S100 calcium binding protein A12	–0,140197724	–0,4103541	Yes
CR1	CR1	complement component (3b/4b) receptor 1 (Knops blood group)	–0,140737161	–0,4087218	Yes
ARRB1	ARRB1	arrestin, beta 1	–0,141240776	–0,4063799	Yes
FOXF2	FOXF2	forkhead box F2	–0,142310947	–0,4061029	Yes
NRG1	NRG1	neuregulin 1	–0,142370075	–0,402846	Yes
CEACAM1	CEACAM1	carcinoembryonic antigen-related cell adhesion molecule 1 (biliary glycoprotein)	–0,142839134	–0,4009717	Yes
FXYD2	FXYD2	FXYD domain containing ion transport regulator 2	–0,143098816	–0,3982038	Yes
RNASE1	RNASE1	ribonuclease, RNase A family, 1 (pancreatic)	–0,144829556	–0,3991322	Yes
CLDN10	CLDN10	claudin 10	–0,145778596	–0,3974389	Yes
FBXO9	FBXO9	F-box protein 9	–0,14605397	–0,3946615	Yes
ETV1	ETV1	ets variant gene 1	–0,147227645	–0,3940731	Yes
LTBP4	LTBP4	latent transforming growth factor beta binding protein 4	–0,14754355	–0,3910055	Yes
AKAP7	AKAP7	A kinase (PRKA) anchor protein 7	–0,149611354	–0,3928299	Yes
NEURL	NEURL	neuralized homolog (Drosophila)	–0,150404543	–0,3925435	Yes
HS3ST2	HS3ST2	heparan sulfate (glucosamine) 3-O-sulfotransferase 2	–0,151680201	–0,3922256	Yes
ITGA2B	ITGA2B	integrin, alpha 2b (platelet glycoprotein IIb of IIb/IIIa complex, antigen CD41)	–0,151895016	–0,3891774	Yes
GLP1R	GLP1R	glucagon-like peptide 1 receptor	–0,153207481	–0,3886317	Yes
TRPM3	TRPM3	transient receptor potential cation channel, subfamily M, member 3	–0,154016465	–0,3870522	Yes
TULP1	TULP1	tubby like protein 1	–0,155865669	–0,3879619	Yes
CEACAM6	CEACAM6	carcinoembryonic antigen-related cell adhesion molecule 6 (non-specific cross reacting antigen)	–0,159271613	–0,3925265	Yes
WDR78	WDR78	WD repeat domain 78	–0,159988225	–0,3902927	Yes
SLC1A7	SLC1A7	solute carrier family 1 (glutamate transporter), member 7	–0,163085416	–0,3942564	Yes

PTGES	PTGES	prostaglandin E synthase	–0,163530424	–0,3910481	Yes
SOX15	SOX15	SRY (sex determining region Y)-box 15	–0,163823128	–0,3877692	Yes
MTSS1	MTSS1	metastasis suppressor 1	–0,165028989	–0,3871855	Yes
GNA11	GNA11	guanine nucleotide binding protein (G protein), alpha 11 (Gq class)	–0,165418416	–0,3843109	Yes
FOXA1	FOXA1	forkhead box A1	–0,166068465	–0,3819272	Yes
PLEKHA5	PLEKHA5	pleckstrin homology domain containing, family A member 5	–0,167007342	–0,3800273	Yes
GNG2	GNG2	guanine nucleotide binding protein (G protein), gamma 2	–0,167509124	–0,3770378	Yes
SNN	SNN	stannin	–0,16781202	–0,3736605	Yes
BIK	BIK	BCL2-interacting killer (apoptosis-inducing)	–0,16848594	–0,3710905	Yes
HLCS	HLCS	holocarboxylase synthetase (biotin-(proprionyl-Coenzyme A-carboxylase (ATP-hydrolysing)) ligase)	–0,168900162	–0,3682568	Yes
TACR1	TACR1	tachykinin receptor 1	–0,169302166	–0,3648427	Yes
GNAQ	GNAQ	guanine nucleotide binding protein (G protein), q polypeptide	–0,170558318	–0,3639326	Yes
APOC2	APOC2	apolipoprotein C-II	–0,170636982	–0,3599788	Yes
VENTXP1	VENTXP1	VENT homeobox (Xenopus laevis) pseudogene 1	–0,171222016	–0,3567075	Yes
GSTA4	GSTA4	glutathione S-transferase A4	–0,171699315	–0,3535513	Yes
ADAM11	ADAM11	ADAM metallopeptidase domain 11	–0,17437616	–0,3557156	Yes
GADD45B	GADD45B	growth arrest and DNA-damage-inducible, beta	–0,174407959	–0,3514153	Yes
RBM12B	RBM12B	RNA binding motif protein 12B	–0,175229147	–0,3495028	Yes
KCNH2	KCNH2	potassium voltage-gated channel, subfamily H (eag-related), member 2	–0,176382512	–0,3469915	Yes
TNFAIP6	TNFAIP6	tumor necrosis factor, alpha-induced protein 6	–0,176826939	–0,3436455	Yes
AAK1	AAK1	AP2 associated kinase 1	–0,180884361	–0,3474871	Yes
MASP2	MASP2	mannan-binding lectin serine peptidase 2	–0,181689501	–0,3445915	Yes
CD80	CD80	CD80 molecule	–0,182976365	–0,342488	Yes
SOX12	SOX12	SRY (sex determining region Y)-box 12	–0,184909075	–0,3420479	Yes
TUBB2B	TUBB2B	tubulin, beta 2B	–0,186026603	–0,3398692	Yes
ARHGEF16	ARHGEF16	Rho guanine exchange factor (GEF) 16	–0,189152211	–0,3411622	Yes
ALPPL2	ALPPL2	alkaline phosphatase, placental-like 2	–0,191411152	–0,3418292	Yes
DSP	DSP	desmoplakin	–0,191655934	–0,3374838	Yes
SIGLEC7	SIGLEC7	sialic acid binding Ig-like lectin 7	–0,192526415	–0,3344477	Yes

TMEM63A	TMEM63A	transmembrane protein 63A	–0,193298638	–0,3313927	Yes
HPCAL4	HPCAL4	hippocalcin like 4	–0,197494239	–0,3351416	Yes
PLAUR	PLAUR	plasminogen activator, urokinase receptor	–0,197547153	–0,3303341	Yes
GNG4	GNG4	guanine nucleotide binding protein (G protein), gamma 4	–0,199557438	–0,3292159	Yes
SLC6A8	SLC6A8	solute carrier family 6 (neurotransmitter transporter, creatine), member 8	–0,200581267	–0,3262347	Yes
GTDC1	GTDC1	glycosyltransferase-like domain containing 1	–0,200864196	–0,3218524	Yes
STRA6	STRA6	stimulated by retinoic acid gene 6 homolog (mouse)	–0,201645434	–0,3184014	Yes
MAST4	MAST4	microtubule associated serine/ threonine kinase family member 4	–0,202050284	–0,3141799	Yes
RAB11B	RAB11B	RAB11B, member RAS oncogene family	–0,20475772	–0,3144545	Yes
FMO2	FMO2	flavin containing monooxygenase 2 (non-functional)	–0,204907328	–0,3097823	Yes
NRP2	NRP2	neuropilin 2	–0,206060007	–0,3066027	Yes
CACNA1I	CACNA1I	calcium channel, voltage-dependent, alpha 1I subunit	–0,20696798	–0,3039076	Yes
DDAH2	DDAH2	dimethylarginine dimethylaminohydrolase 2	–0,207072452	–0,2990554	Yes
TNF	TNF	tumor necrosis factor (TNF superfamily, member 2)	–0,207363307	–0,2946395	Yes
SYT12	SYT12	synaptotagmin XII	–0,207933709	–0,2908434	Yes
LAIR2	LAIR2	leukocyte-associated immunoglobulin-like receptor 2	–0,208619833	–0,2868401	Yes
CDRT1	CDRT1	CMT1A duplicated region transcript 1	–0,2086775	–0,2821384	Yes
ARHGEF15	ARHGEF15	Rho guanine nucleotide exchange factor (GEF) 15	–0,210446477	–0,2797378	Yes
EFHC2	EFHC2	EF-hand domain (C-terminal) containing 2	–0,211410478	–0,2764263	Yes
P2RY4	P2RY4	pyrimidinergic receptor P2Y, G-protein coupled, 4	–0,212946177	–0,2738373	Yes
SAMD14	SAMD14	sterile alpha motif domain containing 14	–0,213386759	–0,2691462	Yes
ATP1B4	ATP1B4	ATPase, (Na+)/K+ transporting, beta 4 polypeptide	–0,213647649	–0,2641952	Yes
ARG1	ARG1	arginase, liver	–0,214615837	–0,2611848	Yes
FLRT2	FLRT2	fibronectin leucine rich transmembrane protein 2	–0,214993075	–0,256771	Yes
ROS1	ROS1	v-ros UR2 sarcoma virus oncogene homolog 1 (avian)	–0,216668233	–0,2558013	Yes

LOC284454	LOC284454	–	–0,216784194	–0,2506462	Yes
KIF5A	KIF5A	kinesin family member 5A	–0,225434408	–0,2587125	Yes
TRIOBP	TRIOBP	TRIO and F-actin binding protein	–0,22909534	–0,2594643	Yes
CAPS	CAPS	calcyphosine	–0,229557142	–0,2545646	Yes
NQO1	NQO1	NAD(P)H dehydrogenase, quinone 1	–0,22982426	–0,2492147	Yes
SYDE1	SYDE1	synapse defective 1, Rho GTPase, homolog 1 (C. elegans)	–0,230991364	–0,2449768	Yes
SCRG1	SCRG1	–	–0,231951192	–0,240525	Yes
TCF20	TCF20	transcription factor 20 (AR1)	–0,234182373	–0,2379194	Yes
NCR2	NCR2	natural cytotoxicity triggering receptor 2	–0,235059142	–0,2330108	Yes
SEMA4G	SEMA4G	sema domain, immunoglobulin domain (Ig), transmembrane domain (TM) and short cytoplasmic domain, (semaphorin) 4G	–0,236343026	–0,2287042	Yes
SHANK2	SHANK2	SH3 and multiple ankyrin repeat domains 2	–0,238577679	–0,2263071	Yes
C1QL1	C1QL1	complement component 1, q subcomponent-like 1	–0,239583328	–0,2217306	Yes
ZBTB7A	ZBTB7A	zinc finger and BTB domain containing 7A	–0,247961909	–0,2251223	Yes
FA2H	FA2H	fatty acid 2-hydroxylase	–0,250364006	–0,2219276	Yes
APAF1	APAF1	apoptotic peptidase activating factor	–0,251709938	–0,2173055	Yes
TCP10L	TCP10L	t-complex 10 (mouse)-like	–0,254182398	–0,2138265	Yes
MAPK10	MAPK10	mitogen-activated protein kinase 10	–0,254703879	–0,2083068	Yes
LIMA1	LIMA1	LIM domain and actin binding 1	–0,257396907	–0,2044951	Yes
MAPK1	MAPK1	mitogen-activated protein kinase 1	–0,257921606	–0,198896	Yes
NCR1	NCR1	natural cytotoxicity triggering receptor 1	–0,260896951	–0,1961387	Yes
HMGA2	HMGA2	high mobility group AT-hook 2	–0,266775638	–0,1951375	Yes
NEUROD2	NEUROD2	neurogenic differentiation 2	–0,267856449	–0,1897371	Yes
HOXA6	HOXA6	homeobox A6	–0,268561751	–0,183939	Yes
HDLBP	HDLBP	high density lipoprotein binding protein (vigilin)	–0,271152973	–0,1790911	Yes
CAMK1G	CAMK1G	calcium/calmodulin-dependent protein kinase IG	–0,275982112	–0,1773559	Yes
EPHB6	EPHB6	EPH receptor B6	–0,281635523	–0,1738337	Yes
CRYM	CRYM	crystallin, mu	–0,281848073	–0,1669477	Yes
EPHB4	EPHB4	EPH receptor B4	–0,282242417	–0,1603687	Yes
CNTD2	CNTD2	cyclin N-terminal domain containing 2	–0,282685757	–0,1539055	Yes
ALPL	ALPL	alkaline phosphatase, liver/bone/kidney	–0,287831008	–0,1505475	Yes

RABEP2	RABEP2	rabaptin, RAB GTPase binding effector protein 2	–0,288437158	–0,1436256	Yes
GRB10	GRB10	growth factor receptor-bound protein 10	–0,291186094	–0,1379035	Yes
CAMK2B	CAMK2B	calcium/calmodulin-dependent protein kinase (CaM kinase) II beta	–0,295495719	–0,1335326	Yes
MTA1	MTA1	metastasis associated 1	–0,2981399	–0,1278924	Yes
DDR1	DDR1	discoidin domain receptor family, member 1	–0,298924267	–0,120712	Yes
EGFR	EGFR	epidermal growth factor receptor (erythroblastic leukemia viral (v-erb-b) oncogene homolog, avian)	–0,300088137	–0,1136297	Yes
SNCA	SNCA	synuclein, alpha (non A4 component of amyloid precursor)	–0,302365273	–0,1075051	Yes
MMP14	MMP14	matrix metallopeptidase 14 (membrane-inserted)	–0,305586994	–0,1017447	Yes
LRRC15	LRRC15	leucine rich repeat containing 15	–0,308051765	–0,0954166	Yes
SLC6A13	SLC6A13	solute carrier family 6 (neurotransmitter transporter, GABA), member 13	–0,308913529	–0,0880532	Yes
MBP	MBP	myelin basic protein	–0,311798096	–0,0822031	Yes
ANKRD26	ANKRD26	ankyrin repeat domain 26	–0,315557986	–0,0763236	Yes
ACTA2	ACTA2	actin, alpha 2, smooth muscle, aorta	–0,322524071	–0,0710961	Yes
ABO	ABO	ABO blood group (transferase A, alpha 1–3-N-acetylgalactosaminyltransferase; transferase B, alpha 1–3-galactosyltransferase)	–0,326877803	–0,0643672	Yes
SASH1	SASH1	SAM and SH3 domain containing 1	–0,330686301	–0,0571641	Yes
SSTR2	SSTR2	somatostatin receptor 2	–0,33428219	–0,0506328	Yes
COL11A2	COL11A2	collagen, type XI, alpha 2	–0,340736002	–0,0443859	Yes
LTBP2	LTBP2	latent transforming growth factor beta binding protein 2	–0,342031747	–0,0362694	Yes
LRFN5	LRFN5	leucine rich repeat and fibronectin type III domain containing 5	–0,342369318	–0,0279544	Yes
SCN10A	SCN10A	sodium channel, voltage-gated, type X, alpha	–0,350856811	–0,0210778	Yes
LDHAL6B	LDHAL6B	lactate dehydrogenase A-like 6B	–0,371172518	–0,0153479	Yes
KLK13	KLK13	kallikrein 13	–0,381830722	–0,0070105	Yes
RHPN1	RHPN1	rhophilin, Rho GTPase binding protein 1	–0,433386177	0,00101381	Yes

Supplementary Table S6: Genes set enrichment analysis revealed a significant overrepresentation of the gene set KEGG_JAK_STAT_SIGNALING_PATHWAY in patients with high Cdh1 expression compared to patients with a low expression (*P* < 0.0001)

Probe	Gene Symbol	Gene Title	Rank Metric Score	Running ES	Core Enrichment
CLCF1	CLCF1	cardiotrophin-like cytokine factor 1	–0,16086638	–0,40052006	Yes
IL26	IL26	interleukin 26	–0,161378995	–0,3912805	Yes
CSF3R	CSF3R	colony stimulating factor 3 receptor (granulocyte)	–0,162488416	–0,3843542	Yes
IFNA8	IFNA8	interferon, alpha 8	–0,164039046	–0,37732828	Yes
CRLF2	CRLF2	cytokine receptor-like factor 2	–0,170205817	–0,38251907	Yes
IL21R	IL21R	interleukin 21 receptor	–0,174330965	–0,38110694	Yes
IFNB1	IFNB1	interferon, beta 1, fibroblast	–0,177312121	–0,37642848	Yes
IFNG	IFNG	interferon, gamma	–0,180473223	–0,37110767	Yes
EPO	EPO	erythropoietin	–0,180844232	–0,35992715	Yes
IL28RA	IL28RA	interleukin 28 receptor, alpha (interferon, lambda receptor)	–0,181333259	–0,3490917	Yes
LEP	LEP	leptin (obesity homolog, mouse)	–0,184159338	–0,3433458	Yes
CBLC	CBLC	Cas-Br-M (murine) ecotropic retroviral transforming sequence c	–0,186741099	–0,33668098	Yes
IL2RA	IL2RA	interleukin 2 receptor, alpha	–0,191764146	–0,3347135	Yes
PIM1	PIM1	pim-1 oncogene	–0,203973159	–0,345704	Yes
IFNA16	IFNA16	interferon, alpha 16	–0,204171255	–0,33296192	Yes
IL22RA2	IL22RA2	interleukin 22 receptor, alpha 2	–0,204459071	–0,32057783	Yes
EPOR	EPOR	erythropoietin receptor	–0,207519859	–0,3143977	Yes
IFNA21	IFNA21	interferon, alpha 21	–0,208058089	–0,30215886	Yes
IL4R	IL4R	interleukin 4 receptor	–0,208607197	–0,28950825	Yes
IL19	IL19	interleukin 19	–0,208873391	–0,2769033	Yes
IL4	IL4	interleukin 4	–0,212494463	–0,26939952	Yes
PRL	PRL	prolactin	–0,214212194	–0,25871056	Yes
SPRY4	SPRY4	sprouty homolog 4 (Drosophila)	–0,21891132	–0,25424576	Yes
IFNA5	IFNA5	interferon, alpha 5	–0,220102981	–0,24167229	Yes
IL24	IL24	interleukin 24	–0,222179636	–0,2305969	Yes
SPRY1	SPRY1	sprouty homolog 1, antagonist of FGF signaling (Drosophila)	–0,240765154	–0,24298848	Yes
CNTFR	CNTFR	ciliary neurotrophic factor receptor	–0,241889313	–0,22914067	Yes

CISH	CISH	cytokine inducible SH2-containing protein	–0,244904652	–0,21735814	Yes
OSM	OSM	oncostatin M	–0,244919658	–0,20162134	Yes
GH1	GH1	growth hormone 1	–0,24963221	–0,19003707	Yes
IL23A	IL23A	interleukin 23, alpha subunit p19	–0,250638574	–0,17518784	Yes
IL21	IL21	interleukin 21	–0,251465917	–0,16009718	Yes
AKT3	AKT3	v-akt murine thymoma viral oncogene homolog 3 (protein kinase B, gamma)	–0,260078698	–0,15311278	Yes
PRLR	PRLR	prolactin receptor	–0,267037302	–0,14292021	Yes
MPL	MPL	myeloproliferative leukemia virus oncogene	–0,272604197	–0,13067569	Yes
CREBBP	CREBBP	CREB binding protein (Rubinstein-Taybi syndrome)	–0,275879741	–0,116275415	Yes
CSF2	CSF2	colony stimulating factor 2 (granulocyte-macrophage)	–0,276551753	–0,09894542	Yes
IL20RA	IL20RA	interleukin 20 receptor, alpha	–0,28004536	–0,082834214	Yes
IL10RB	IL10RB	interleukin 10 receptor, beta	–0,294243962	–0,073215336	Yes
IFNA6	IFNA6	interferon, alpha 6	–0,303986788	–0,059707392	Yes
GHR	GHR	growth hormone receptor	–0,325781286	–0,04875241	Yes
STAT5B	STAT5B	signal transducer and activator of transcription 5B	–0,331452936	–0,029087119	Yes
SOS2	SOS2	son of sevenless homolog 2 (Drosophila)	–0,334231466	–0,008929544	Yes
IFNA10	IFNA10	interferon, alpha 10	–0,339182347	0,01110688	Yes

Supplementary Table S7: Genes set enrichment analysis revealed a significant overrepresentation of the gene set SCHUHMACHER_MYC_TARGETS_UP in patients with low Cdh1 expression compared to patients with a high expression (*P* = 0.002)

Probe	Gene Symbol	Gene Title	Rank Metric Score	Running ES	Core Enrichment
MYC	MYC	v-myc myelocytomatosis viral oncogene homolog (avian)	0,255174637	0,039218597	Yes
TARBP1	TARBP1	Tar (HIV-1) RNA binding protein 1	0,245373219	0,08355752	Yes
CTPS	CTPS	CTP synthase	0,239183694	0,12721579	Yes
RCC1	RCC1	regulator of chromosome condensation 1	0,235875025	0,17155375	Yes
EXOSC2	EXOSC2	exosome component 2	0,212953702	0,206281	Yes
SLC39A6	SLC39A6	solute carrier family 39 (zinc transporter), member 6	0,203168482	0,24257496	Yes
DCUN1D4	DCUN1D4	DCN1, defective in cullin neddylation 1, domain containing 4 (S. cerevisiae)	0,20088844	0,27968228	Yes
PRPS2	PRPS2	phosphoribosyl pyrophosphate synthetase 2	0,188228428	0,31025803	Yes
TMEM97	TMEM97	transmembrane protein 97	0,181349173	0,34158167	Yes
ATP1B3	ATP1B3	ATPase, Na+/K+ transporting, beta 3 polypeptide	0,167048037	0,36599854	Yes
SORD	SORD	sorbitol dehydrogenase	0,156934127	0,39023468	Yes
AKAP1	AKAP1	A kinase (PRKA) anchor protein 1	0,155322924	0,418594	Yes
SLC16A1	SLC16A1	solute carrier family 16, member 1 (monocarboxylic acid transporter 1)	0,15338476	0,44695824	Yes
AHCY	AHCY	S-adenosylhomocysteine hydrolase	0,139499024	0,46369025	Yes
MTHFD1	MTHFD1	methylenetetrahydrofolate dehydrogenase (NADP+ dependent) 1, methenyltetrahydrofolate cyclohydrolase, formyltetrahydrofolate synthetase	0,126711503	0,47573838	Yes
CTSC	CTSC	cathepsin C	0,125715002	0,49864107	Yes
UCK2	UCK2	uridine-cytidine kinase 2	0,108520187	0,50116897	Yes
MEST	MEST	mesoderm specific transcript homolog (mouse)	0,108244635	0,5215527	Yes
TFRC	TFRC	transferrin receptor (p90, CD71)	0,101884656	0,53410983	Yes
RABEPK	RABEPK	Rab9 effector protein with kelch motifs	0,101412199	0,55312884	Yes

ACSL1	ACSL1	acyl-CoA synthetase long-chain family member 1	0,097003475	0,5660035	Yes
TRAP1	TRAP1	TNF receptor-associated protein 1	0,090697132	0,5758042	Yes
SLC39A14	SLC39A14	solute carrier family 39 (zinc transporter), member 14	0,089404106	0,5912862	Yes
RPIA	RPIA	ribose 5-phosphate isomerase A (ribose 5-phosphate epimerase)	0,085448854	0,60264474	Yes
VARS	VARS	valyl-tRNA synthetase	0,080577493	0,6107036	Yes
MGST1	MGST1	microsomal glutathione S-transferase 1	0,079640403	0,6251357	Yes
POLD2	POLD2	polymerase (DNA directed), delta 2, regulatory subunit 50kDa	0,077954493	0,6381856	Yes
IMPDH2	IMPDH2	IMP (inosine monophosphate) dehydrogenase 2	0,059360199	0,62148374	No
SLC20A1	SLC20A1	solute carrier family 20 (phosphate transporter), member 1	0,038450092	0,5889403	No
PEBP1	PEBP1	phosphatidylethanolamine binding protein 1	0,001090628	0,50846684	No
DDX10	DDX10	DEAD (Asp-Glu-Ala-Asp) box polypeptide 10	-2,31E–04	0,50501645	No
RANBP1	RANBP1	RAN binding protein 1	–0,00286241	0,49901024	No
CEBPZ	CEBPZ	CCAAT/enhancer binding protein zeta	–0,024270432	0,44797704	No
CYCS	CYCS	cytochrome c, somatic	–0,034888688	0,4208074	No
CAD	CAD	carbamoyl-phosphate synthetase 2, aspartate transcarbamylase, and dihydroorotase	–0,042338245	0,4069135	No
PPAT	PPAT	phosphoribosyl pyrophosphate amidotransferase	–0,069140054	0,33747706	No
IARS	IARS	isoleucine-tRNA synthetase	–0,093527265	0,27268925	No
PAICS	PAICS	phosphoribosylaminoimidazole carboxylase, phosphoribosylaminoimidazole succinocarboxamide synthetase	–0,154700935	0,116791725	No
NEFH	NEFH	neurofilament, heavy polypeptide 200kDa	–0,162272066	0,12912886	No
AUH	AUH	AU RNA binding protein/ enoyl-Coenzyme A hydratase	–0,167197838	0,148208	No
LRP8	LRP8	low density lipoprotein receptor-related protein 8, apolipoprotein e receptor	–0,175224364	0,1617661	No
EXOSC7	EXOSC7	exosome component 7	–0,212952539	0,12604512	No

Supplementary Table S8: IC50 of proTAME

IC50 proTAME	
**Cell type**	**IC50 (μM)**
LP-1	12,1
RPMI-8226	6,4
NCI-H929	10,5
U266	7,1
OPM-2	7,5
JJN3	4,8
Patient 1	7,8
Patient 2	4,9
Patient 3	2,8
Patient 4	20,3
Patient 5	8,3
Patient 6	12.0
Patient 7	7,8
PBMC	73,6

Different cells were treated with 3, 6, 12, 24 μM proTAME and after 24 h the viability was measured with the CellTiter-Glo assay. The IC50 was calculated using GraphPad Prism 5.01 software at the end of the sentence.

Supplementary Table S9: patient characteristics and previous treatment

Patient	ISS	Isotype	Previous treatment	Cytogenetics
Patient 1	1	Light chain κ and IgD	VRD, ASCT, R	del17p, t (4;14)
Patient 2	3	IgGκ	New	del17p, t (4;14)
Patient 3	1	IgGλ	VD, ASCT	Normal
Patient 4	3	IgGκ	New	Normal
Patient 5	1	IgGκ	New	Normal
Patient 6	2	Light chain κ	RDI	Del13q
Patient 7	1	IgGκ	KRD, ASCT, KRD, R	Normal

ISS: International Stating System at diagnosis; κ: kappa; λ: lambda; ASCT: autologous stem cell transplantation; V: velcade; R: revlimid; D: dexamethasone; I: ixazomib; K: kyprolis
